# The application of metal artifact reduction methods on computed tomography scans for radiotherapy applications: A literature review

**DOI:** 10.1002/acm2.13255

**Published:** 2021-05-03

**Authors:** Sathyathas Puvanasunthararajah, Davide Fontanarosa, Marie‐Luise Wille, Saskia M. Camps

**Affiliations:** ^1^ School of Clinical Sciences Queensland University of Technology Brisbane QLD Australia; ^2^ Centre for Biomedical Technologies Queensland University of Technology Brisbane QLD Australia; ^3^ School of Mechanical Medical & Process Engineering Faculty of Engineering Queensland University of Technology Brisbane QLD Australia; ^4^ ARC ITTC for Multiscale 3D Imaging, Modelling, and Manufacturing Queensland University of Technology Brisbane QLD Australia; ^5^ EBAMed SA Geneva Switzerland

**Keywords:** computed tomography, metal artifact reduction, metal artifacts, radiotherapy

## Abstract

Metal artifact reduction (MAR) methods are used to reduce artifacts from metals or metal components in computed tomography (CT). In radiotherapy (RT), CT is the most used imaging modality for planning, whose quality is often affected by metal artifacts. The aim of this study is to systematically review the impact of MAR methods on CT Hounsfield Unit values, contouring of regions of interest, and dose calculation for RT applications. This systematic review is performed in accordance with the PRISMA guidelines; the PubMed and Web of Science databases were searched using the main keywords “metal artifact reduction”, “computed tomography” and “radiotherapy”. A total of 382 publications were identified, of which 40 (including one review article) met the inclusion criteria and were included in this review. The selected publications (except for the review article) were grouped into two main categories: commercial MAR methods and research‐based MAR methods. Conclusion: The application of MAR methods on CT scans can improve treatment planning quality in RT. However, none of the investigated or proposed MAR methods was completely satisfactory for RT applications because of limitations such as the introduction of other errors (e.g., other artifacts) or image quality degradation (e.g., blurring), and further research is still necessary to overcome these challenges.

**Abbreviations**
AAA: anisotropic analytical algorithm
ALIR: augmented likelihood image reconstruction
AXB: Acuros External Beam
CNNs: convolutional neural network
CT: computed tomography
CT_art_: CT scan with metal artifacts
CT_cor_: CT scan after metal artifact reduction
CT_ref_: CT scan without metal artifacts
DAH: dose area histogram
DL‐MAR: deep learning‐based metal artifact reduction
DSC: Dice similarity coefficient
GANs: generative adversarial networks
GTV: gross tumor volume
H&N: head and neck
HD: Hausdorff distance
HU: Hounsfield units
iMAR: iterative metal artifact reduction
IMRT: intensity‐modulated radiation therapy
kVCT: kilovoltage computed tomography
kVp: kilovoltage peak
LI: linear interpolation
MAR: metal artifact reduction
MBIR: model‐based iterative reconstruction
MDT: metal deletion technique
MV: megavoltage
MVCBCT: megavoltage con‐beam computed tomography
MVCT: megavoltage computed tomography
N‐MAR: normalized metal artifact reduction
OARs: organs at risk
O‐MAR: orthopedic metal artifact reduction
PSNR: peak signal‐to‐noise ratio
PTV: planning target volume
RL‐ARCNN: residual learning‐artifact reduction convolutional neural network
ROI: region of interest
RT: radiotherapy
SBRT: stereotactic body radiation therapy
SEMAR: single energy metal artifact reduction
SSIM: structural similarity
STD: standard deviation
STR: simple threshold replacement
TPS: treatment planning system
US: ultrasound
VMAT: volumetric arch therapy
WET: water equivalent thickness
%GP: gamma passing rate

## 1 INTRODUCTION

Radiotherapy (RT) is one of the primary curative treatment options for different types of cancer, for example, cancers of head and neck, prostate, cervix, breast, as well as sarcomas. RT aims to deliver therapeutic ionizing radiation dose to a treatment target, while sparing healthy organs at risk (OARs) as much as possible. The therapeutic radiation dose is delivered to the target using beams produced by a clinical linear accelerator (LINAC) in external beam radiation therapy (EBRT), while in brachytherapy radioactive sources invasively placed near or inside the target are used. Typically, RT workflows include a simulation stage and a treatment delivery stage. During the treatment planning process at the simulation stage, computed tomography (CT) scans serve as a primary source of anatomical information to identify and delineate the target and OARs. In addition, they are used to calculate the electron densities which are derived from the Hounsfield unit (HU) values of those CT scans. This electron density information in combination with the delineations of the anatomical structures is used to calculate the therapeutic radiation dose.

**Fig. 1:**
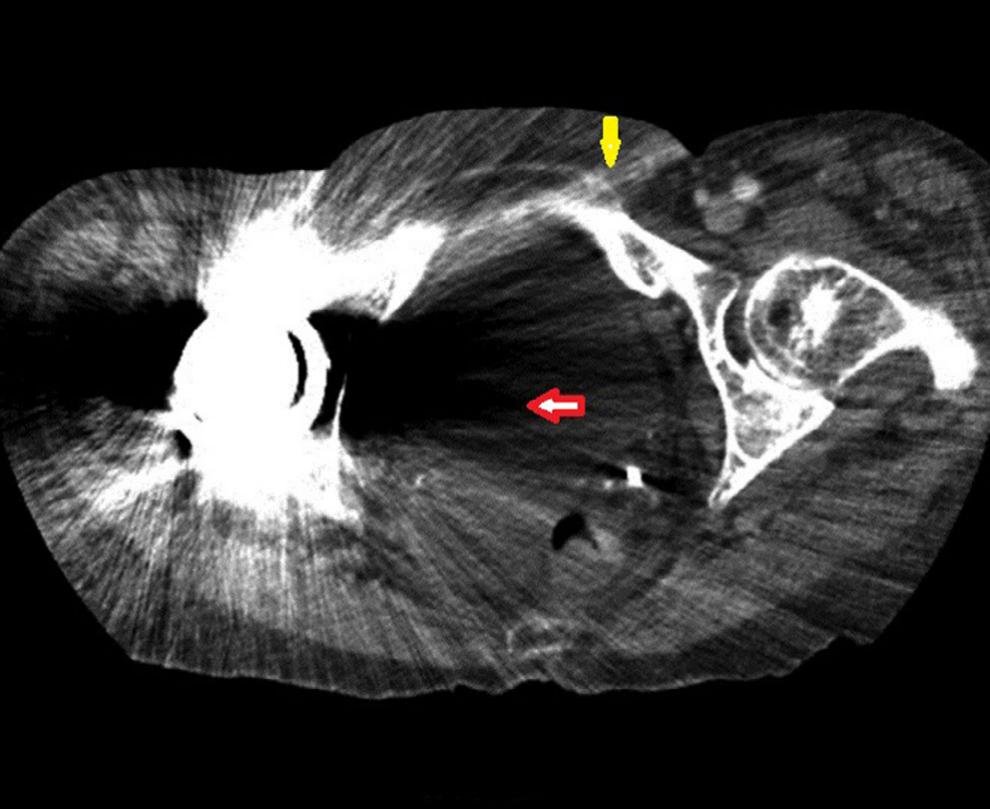
CT scan with artifacts induced by a unilateral hip implant with the appearance of bright streaks (indicated by yellow arrow) and dark streaks (indicated by red arrow).

CT scans with insufficient quality may greatly affect the treatment planning process, potentially resulting in the target receiving insufficient dosage and/or extra toxicity to the OARs. Metal implants or metal components inside the body of the patient can induce errors during the CT reconstruction, which appear as artifacts on the resulting CT scans. These metal artifacts are typically bright and/or dark streaks (see Fig. 1) and are produced by beam hardening, photon starvation, edge gradient effect, scatter, or their combination.^1^, ^2^ The degree of metal artifacts mainly depends on the atomic number, density, size, and shape of this metal component as well as its orientation with respect to the CT scan plane.^3^, ^4^ Among others, dental implants or dental fillings in the head and neck (H&N) area, bilateral or unilateral metal prostheses in the hip region, and metal screws in the spine produce a large amount of metal artifacts and, thus, significantly deteriorate the quality of CT scans.^5^, ^6^, ^7^

Dark streaks near the metal components result from highly attenuated polychromatic x‐ray beams, which become for this reason harder.^8^ Because of this, insufficient photons reach the CT detectors (photon starvation), resulting in large statistical errors in data acquisition, which induce fine bright and dark streaks along the direction of highest attenuation^7^, ^8^. As a consequence, the appearance of these streak‐shaped metal artifacts adversely affects the accuracy of organ contouring and the electron density calculation. This can eventually result in errors in planned radiation dose distributions and particle range measurements in photon and particle beams, respectively.^9^, ^10^

In the literature, several papers have been published on algorithms which perform metal artifact reduction (MAR) on CT scans. The working principle of traditional MAR algorithms may be categorized into three overall approaches: image inpainting techniques,^11^ sinogram inpainting techniques,^12^ and model‐based iterative reconstruction (MBIR) techniques^13^ or their combination. The image inpainting techniques are applied to already reconstructed CT scans and they replace artifact corrupted CT pixels with good‐estimated values. The sinogram inpainting techniques follow a similar principle, but are used on projection data (sinograms) instead of on reconstructed CT slices. Finally, MBIR techniques are advanced CT reconstruction techniques which use probabilistic forward and backward models to reduce error propagations during CT reconstruction.^14^ Recently, thanks to the increasing availability of computational resources, very promising results in the field of medical imaging have been produced using machine learning (and in particular its subset deep learning),^15^, ^16^, ^17^, ^18^ including metal artifact reduction in CT scans.^19^, ^20^ For example, the performance of convolutional neural networks (CNNs) has been assessed in combination with sinogram inpainting for artifact correction.^19^, ^21^ The deep learning techniques are powerful in learning and capturing the detailed features and patterns of the metal artifacts.

In general, the application of a MAR method on a CT scan with artifacts (CT_art_) results in the creation of a corrected CT scan (CT_cor_) on which the impact of the artifacts is reduced, either in terms of image quality or dosimetric outcome on the treatment. To measure the effectiveness of the methods, several different metrics have been introduced in the literature to compare CT_art_ and CT_cor_. Image quality metrics proposed include visual inspection, quantification of HU values, artifact index,^22^ contrast‐to‐noise ratio (CNR), signal‐to‐noise ratio (SNR), peak SNR (PSNR), structural similarity (SSIM), Hausdorff distance (HD),^23^ and the Dice similarity coefficient (DSC).^24^, ^25^, ^26^ To evaluate the dosimetric impact, instead, the calculated dose distributions on CT_art_ and CT_cor_ for the target and OARs provided by a treatment planning system (TPS) can be compared. Various dose metrics can be used to express the dosimetric impact, including gamma (γ) index,^27^, ^28^ dose‐area histogram (DAH),^29^ quantifications of D_90%_, D_100%_, V_100%_, and V_150%_,^30^ and therapeutic range calculation (in particular, water equivalent thickness (WET)^31^ in particle therapy).

A topical review article has been previously published by Giantsoudi et al. on the evaluation of the dosimetric effects of metal artifacts on treatment planning and the potential dosimetric improvements resulting from the application of various MAR methods.^10^ The article addressed the impact of sinogram inpainting and MBIR on the dose distributions, mainly focusing on research‐based MAR methods while, among the commercially available MAR methods, only the Orthopaedics Metal Artifact Reduction (O‐MAR (Philips Health System, Cleveland, USA)) algorithm was reported. Instead, this systematic review article aims to include all MAR methods which have been investigated or proposed for RT applications in the last 5 yrs at the time of publication (2015–2020). These methods include commercial MAR methods and research‐based MAR methods based on either traditional algorithms or deep learning. In addition, our review extensively reports not only the works on dosimetric impact of the methods but also on the ones evaluating the effects on organ contouring, and image quality and HU restoration for RT applications.

## 2 METHODS

### 2.A Literature search

**Fig. 2:**
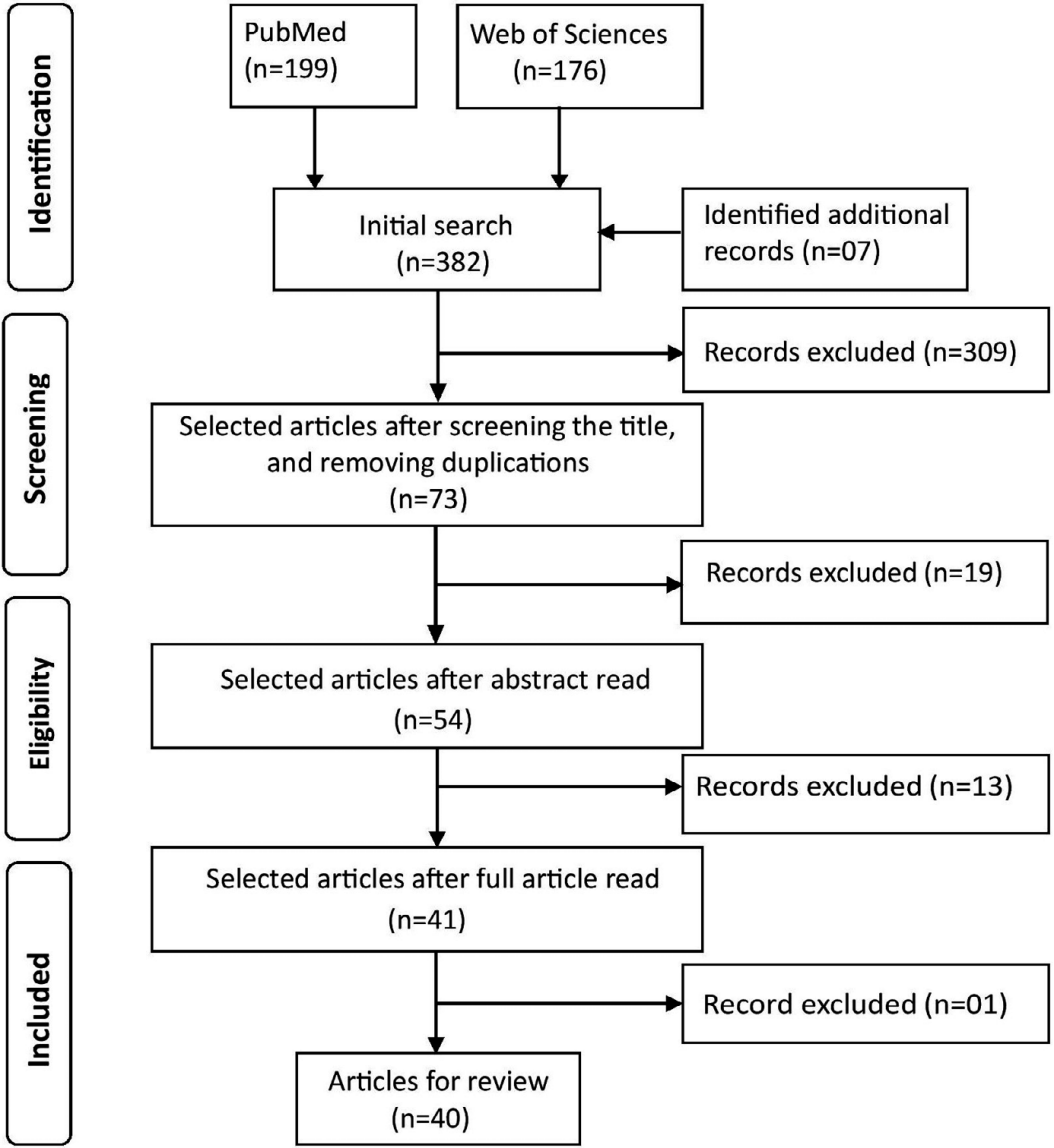
PRISMA flow chart for the selection of the articles for this review.

The systematic review search was performed in accordance with the Preferred Reporting Items for Systematic Reviews and Meta‐Analyses (PRISMA) guidelines.^32^, ^33^ A comprehensive electronic search from the databases of PubMed® (U.S. National Library of Medicine, USA) and Web of Science (Clarivate Analytics, USA) was performed in October 2020. Combinations and synonyms of the main keywords “metal artifact reduction,” “computed tomography,” and “radiotherapy” were used. The search was limited to the English language and to the last 5 yrs. Initially, title and abstract of the identified articles were read to screen their suitability for the selection. Then, the full texts were read from the selected articles to check their eligibility for inclusion. Finally, a manual search was performed using the list of references of the included articles to find any additional data missed by the initial database searches (see Fig. 2).

### 2.B Inclusion and exclusion criteria

An article was considered if it investigated the use of one or multiple MAR methods on CT scans for RT applications, with the exclusion of dual‐energy CT (DECT), dental cone‐beam CT (CBCT), C‐arm CT, spectral CT, micro‐CT, or photoacoustic CT. Also, editorial commentaries and book chapters were excluded.

## 3 RESULTS

A total of 40 full‐text publications were selected for this systematic review, including one review article, as mentioned in the Introduction section. The selected publications (except the review article) have further been categorized into application of commercial methods (n = 25), and application of research‐based MAR methods (n = 14). The category of commercial methods includes the articles on commercial MAR algorithms (n = 21) and TPS‐based density correction (n = 4). Research‐based MAR methods include the articles on traditional MAR algorithms (n = 11) and deep learning‐based MAR algorithms (n = 3).

### 3.A Commercial MAR methods

Commercial MAR methods are available on CT scanners or on the TPS to reduce metal artifacts on CT_art_ for RT applications. The algorithms implemented directly on CT scanners use a sinogram inpainting technique with iterative reconstruction and include: O‐MAR (Philips Health System, Cleveland, USA),^34^ iterative metal artifact reduction (iMAR [Siemens Healthcare, Forchheim, Germany]),^35^ smart metal artifact reduction (Smart MAR [General Electric Healthcare, Chicago, IL, USA]),^36^ and single‐energy metal artifact reduction (SEMAR [Canon/Toshiba Medical Systems, Otawara, Japan]).^37^ These algorithms work on projection data (projection‐based MAR algorithms) and they typically use an image‐based metal segmentation method as a starting point.^7^ Their basic concept is to detect and segment the corrupted projection data which corresponds to the metal components. Subsequently, the corrupted data are replaced by estimated corrected values.

**Fig. 3:**
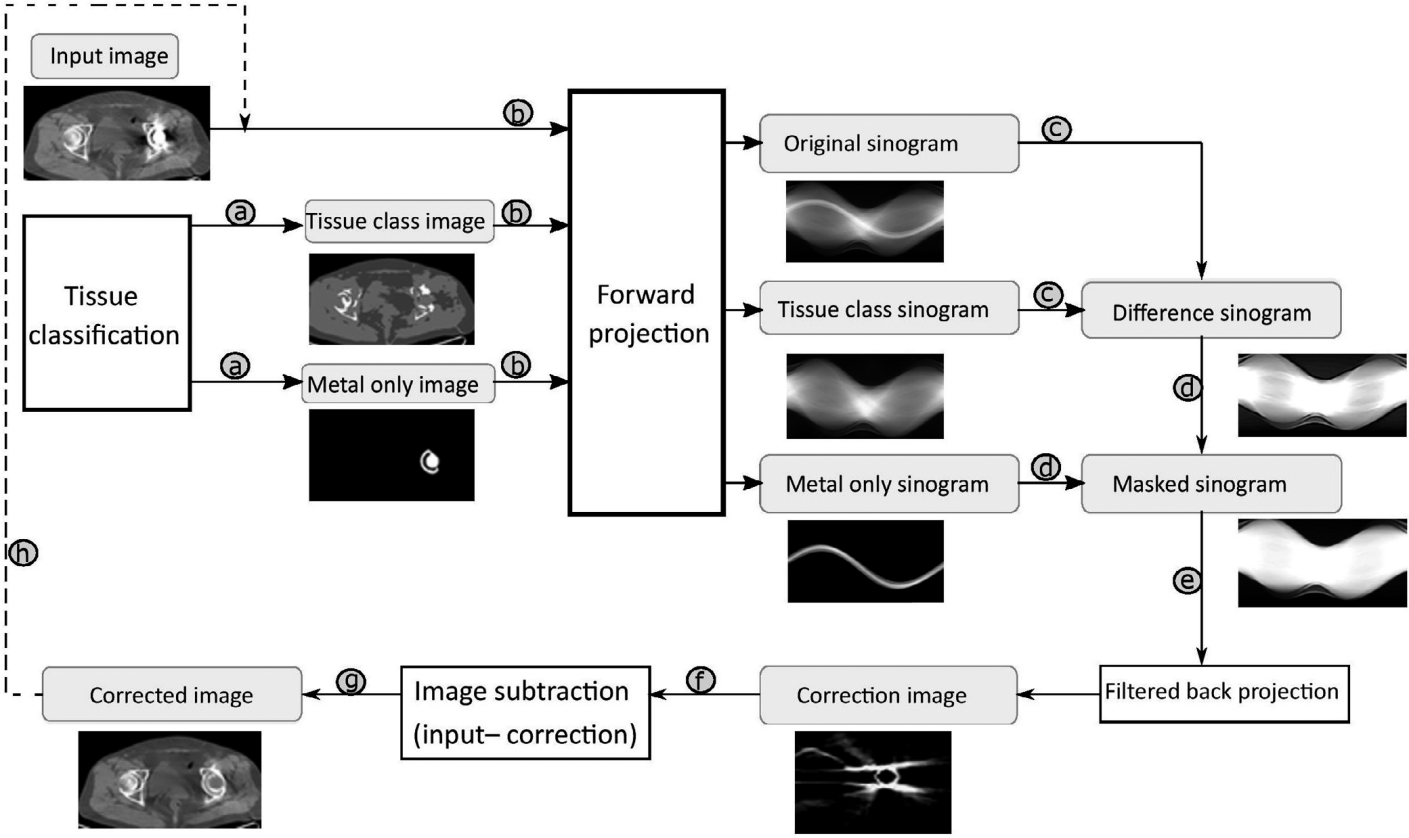
Working principle of the O‐MAR algorithm. Starting from tissue classification, O‐MAR produces original, tissue class, and metal‐only sinograms (a and b). Subtraction (c) of the tissue class sinogram from the original sinogram results in a difference sinogram. Then, the metal‐only sinogram is used to mask (d) the difference sinogram and a correction image is produced after filtered back projection (e). Subtraction of the correction image from the input (f and g) produces a corrected image which then undergo (h) in order to apply further corrections.

Several studies have investigated the applicability of commercial MAR algorithms, especially O‐MAR, in RT. These commercial MAR algorithms are not openly accessible; however, O‐MAR is chosen here for a general introduction to the working principles of all of them. The O‐MAR algorithm is optimized for orthopedic metal implants and uses an iterative projection modification method to reduce the artifacts (see Fig. 3). First, a tissue classification process is performed which assigns tissue labels to all the pixels of an original input image (see arrows ‘a’ in Fig. 3). Two separate images are, thus, produced: one including only tissues and one only the metal component. Subsequently, both segmented images (tissue classified and metal only) and the original input image are forward projected to make their respective sinograms (see arrows ‘b’ in Fig. 3). After the sinograms are made, the tissue classified sinogram is subtracted from the original sinogram (see arrows ‘c’ in Fig. 3) resulting in the creation of a difference sinogram. Then, the metal‐only sinogram is used as a mask to remove the nonmetal pixels from the difference sinogram (see arrows ‘d’ in Fig. 3) and a mask sinogram is created. A correction image is then produced by filtered back projection of the mask sinogram (see arrow ‘e’ in Fig. 3), and subtraction of the correction image from the original input image is created (see arrows ‘f’ and ‘g’ in Fig. 3). Finally, the corrected image is used as an input in an iterative loop for further correction (see arrow ‘h’ in Fig. 3).

A density correction or a density override method on the TPS can also be used to reduce the metal artifacts on CT_art_. When these approaches are used, regions corrupted by metal artifacts and metal regions are identified manually through contouring on CT_art_. Then, a manual density override or density correction is performed by replacing the metal artifacts commonly with the density of water or by replacing the physical density of a metal implant by the appropriate value.^38^, ^39^

#### 3.A.1 Evaluation of HU values restoration

The capability of the commercial MAR algorithms to improve the HU values for RT applications was evaluated by several studies. Generally, during the HU measurements for an anatomical region on a CT scan, a 10%–20% variations in HU values are expected. For example, at routine CT scans, the HU value of water is set to 0 HU. The HU value of air is approximately −1000 HU and bone is around 1000 HU, while soft tissue HU values range from 20 HU to 30 HU.^40^ Table 1 provides an overview of the studies which investigate the HU value improvements.

**Table 1:** Summary of the studies which investigated commercial MAR algorithms for HU value restoration for RT applications.

Author	MAR	Images	Metals	CT scans	ROI; [Reference HU value (Mean)]	Measurements	Results	Findings
Kwon et al.^41^	O‐MAR	Clinical (H&N, open mouth), (n = 3)	Dental implants	CT_art_ & CT_cor_	Air in outer cavity; [−1000 HU]	Mean HU	‐238.7 HU & ‐441.8 HU	O‐MAR increased the accuracy of HU values
Bär et al.^42^	iMAR	Phantom	Al, Ti & SS	CT_ref_ vs CT_cor_	Tissue equivalent substitutes; [Not reported]	Absolute HU difference	44 HU	iMAR restored the HU values independent of the metal density
Axente et al.^35^	iMAR	Phantom	SS with varying diameters	CT_ref_ vs CT_cor_	Multiple places	Absolute HU difference	25 HU	iMAR restored the HU values independent of the metal size and configuration
Maerz et al.^43^	iMAR	Phantom	Dental implants	(CT_art_ vs CT_ref_) & (CT_cor_ vs CT_ref_)	Tissue; [Not reported] Metal inserts; [Not reported]	Mean HU difference	283 HU & 33 HU 1006 & 408 HU	iMAR improved the accuracy of HU value measurements
Guilfoile et al.^44^	Smart MAR	Phantom	Samarium cobalt magnet	(CT_art_ vs CT_ref_ ) & (CT_cor_ vs CT_ref_)	Ipsilateral lung Spinal bone [Not reported]	Mean HU	29HU & 2HU 21HU & 8HU	Application of Smart MAR improved the HU values restoration
Huang & Kohli^45^	Smart MAR	Phantom	SS	CT_cor_ vs CT_art_	Multiple places	STD reduction	9.1 HU	Smart MAR reduced the STD of HU values
Inal & Sarpün^46^	Smart MAR	Phantom	Lead	CT_art_ & CT_cor_	Area close to the metal edge; [Around 0 HU]	Mean HU	‐862 HU & −185 HU	Smart MAR improved the accuracy of HU value measurements
Murazaki et al.^47^	SEMAR	Phantom	Metal inserts	CT_art_ & CT_cor_	Muscle substitute; [25.9 HU]	Mean HU	−281.8 HU & 26.1 HU	SEMAR restored the HU values with high level of accuracy
Miki et al.^48^	SEMAR	Phantom	Metal screws	(CT_art_ vs CT_ref_ ) & (CT_cor_ vs CT_ref_)	Severe artifact region; [Not reported]	Mean (± STD) HU	‐79.5 (± 97.2) HU & ‐1.4 (± 19.5) HU	SEMAR improved the HU value measurements accuracy

HU Hounsfield units, H&N Head and neck, Al Aluminum, Ti Titanium, SS Stainless steel, CTart CT scans with artifacts, CTcor Corrected CT scans, CTref Reference CT scans, vs versus.

Kwon et al. studied the impact of O‐MAR on both clinical H&N CT scans with dental implants, and on CT scans of a custom‐made phantom with aluminum (Al), titanium (Ti), zirconium (Zr), and chromium (Cr) metal implants.^41^ The CT_cor_ after O‐MAR application showed HU values closer (not quantified) to the actual values, while the noise on clinical H&N CT scans was reduced. For the phantom, a comparison between CT_cor_ after O‐MAR application and CT scans without metal implants (CT_ref_) did not show significant differences while significant differences (*P* < 0.05) were observed between CT_art_ and CT_ref_.

For the evaluation of iMAR by Bär et al., a Gammex 467 (Gammex, Middleton, WI, USA) phantom was used with several tissue‐equivalent inserts, such as lung, adipose tissue, breast, liver, and bone.^42^ The phantom was scanned with and without Al, Ti, and stainless steel (SS) implants. This study revealed that iMAR application improved the HU accuracy on CT_cor_ compared to CT_art_. For example, iMAR approximated the HU values for tissue‐equivalent substitutes in the phantom up to ±44 HU compared to CT_ref_. A similar finding was found by the authors for H&N clinical CT scans with dental fillings and/or implants. Moreover, they stated that iMAR corrected the HU values independently of the metal density. In another study by Axente et al., a standard electron density phantom (CIRS, Model 062MA, Norfolk, VA, USA) was used with multiple inserts, such as plastic water, bone, muscle, adipose tissue, breast, bone, lung, and liver.^35^ The phantom with and without SS inserts with varying diameters was CT scanned. It was shown that the CT_cor_ after iMAR application restored the HU values well and it had absolute differences of less than 25 HU compared to CT_ref_. Nevertheless, residual HU errors (not quantified) were observed on the resulting CT_cor_. Furthermore, the study mentioned that iMAR restored the HU independently of the metal component’s size and configuration. Maerz et al. reported, in their dental cylindrical phantom study, that the use of iMAR reduced the average HU deviation, from 1006 HU on CT_art_ to 408 HU in the area which included the metal inserts, and from 283 HU on CT_art_ to 33 HU in tissue areas.^43^

Guilfoile et al. investigated the ability of Smart MAR to restore the HU values. To this end, a breast expander consisting of a samarium cobalt magnet was placed on an adult male phantom (CIRS, Model ATOM 701‐B, Norfolk, VA, USA).^44^ The authors compared the mean HU value on CT_ref_ (not given) with both CT_art_ and CT_cor_ after Smart MAR application. The results showed a reduction in the HU value difference from 29 HU to 3 HU in ipsilateral lung, and from 21 HU to 8 HU in bone close to the metal implant, respectively. In addition, small differences in mean HU values were measured in regions that were further away from the implant. In a study with a Catphan® 504 phantom (The Phantom Laboratory, NY, USA) with a SS implant, Huang and Kohli found that Smart MAR substantially reduced the metal artifacts.^45^ On average, the standard deviation (STD) was reduced by 9.1 HU on CT_cor_ after Smart MAR application in comparison with CT_art_. Another study used a custom‐made water equivalent phantom with lead implants and concluded that Smart MAR application improved the mean HU value in a region of interest (ROI) close to a lead implant from −862 HU on CT_art_ to −185 HU on CT_cor_.^46^ This low HU value measurement for water on CT_art_ (water usually is around 0 HU) resulted from the severe dark streak artifact from the metal implant. The application of iMAR on CT_art_ reduced the severity of the dark streaks and, therefore, improved the HU value of water.

A study by Murazaki et al. investigated the results of SEMAR application on HU value measurements accuracy.^47^ The authors made use of a standard electron density phantom (CIRS, Model 062A, Norfolk, VA, USA) with and without Ti implants. To simulate the different tissue types, plugs with different densities were inserted into the phantom, such as muscle plugs and soft tissue plugs. They found that, in the muscle plug, the mean HU value on CT_ref_, CT_art_, and CT_cor_ after SEMAR application were 25.9 HU, −281.8 HU, and 26.1 HU, respectively. A similar pattern of HU value measurements was observed in other tissue plugs. In an experiment by Miki et al., an anthropomorphic head phantom was CT scanned with and without the insertion of metal crews.^48^ In areas with severe artifacts, HU value measurements on both CT_art_ and CT_cor_ after SEMAR application were compared with CT_ref_. This resulted in differences of −79.5 ± 97.2 HU (mean ± STD) and −1.4 ± 19.5 HU (mean ± STD), respectively. The HU value of CT_ref_ was not reported. The CT_cor_ after SEMAR application brought the HU values closer to the reference values.

In general, the majority of the studies used phantoms to investigate the capability of the commercial MAR algorithm to restore HU values and they concluded that the investigated commercial MAR algorithms improved the accuracy of HU value measurements. Nevertheless, residual HU errors were observed on CT_cor_, either underestimating or overestimating the correct HU values. Underestimation resulted from incomplete correction of dark streak artifacts, while overestimation resulted from incomplete correction of bright streaks on CT_cor_.

#### 3.A.2 Evaluation of organ contouring

The commercial MAR algorithms have been also evaluated on their ability to improve organ contouring in RT (see Table 2). A study by Sillanpaa et al. evaluated the easiness of contouring of the parotid gland on clinical H&N CT scans with dental fillings.^49^ The contouring of the parotid was performed on CT_art_ and CT_cor_ after O‐MAR application. During the contouring, 79% of the CT_cor_ and 11% of the CT_art_ were classified as easy to contour. Furthermore, the authors calculated the dice similarity coefficient (DSC) to assess the contouring interobserver variability. The perfect match between two contours, on the same anatomy, would result in the highest DSC value of 1, while the lowest DSC value of 0 represents no overlap of two contoured structures. The average DSC for the parotid contouring was 0.775 ± 0.045 (mean ± STD) on both CT_cor_ (after O‐MAR application) and CT_art_. These clinical H&N CT scans had large amounts of small dental fillings on either side of jaw, which might cause incomplete correction of metal artifacts. The authors suggested that this was one of the reasons for classifying 11% of the CT_art_ as easy to contour.

**Table 2:** Summary of the studies which investigated the commercial MAR algorithms for organ contouring for RT.

Author	MAR	Images; No. of sample (n)	Implants	CT scans	ROI	Measurements	Results	Findings
Sillanpaa et al.^49^	O‐MAR	Clinical (H&N); (n = 28)	Dental implants	CT_art_ & CT_cor_ CT_cor_ vs CT_art_	Parotid gland	Contouring easiness (%) DSC difference [Mean ± STD]	79% and 11% 0.775 ± 0.045	O‐MAR made contouring easy with less interobserver variability
Andersson et al.^50^	O‐MAR & iMAR	Clinical (H&N); (n = 30)	Dental implants	CT_art_ vs CT_cor_	Whole images	Visual grading score	Refer to fig. 8 in Ref. [50]	O‐MAR and iMAR improved the image quality for accurate contouring
Kohan et al.^51^	O‐MAR	Clinical (H&N); (n = 11)	Dental implants	CT_art_, CT_cor_ & CT_ref_	Masseter muscle, tongue, jaw, upper maxilla, & major pectoralis muscle	ICC All reviewers: Experienced reviewers:	0.884,0.971 & 0.989 (w/o) 0.903,0.948 & 0.985 0.934,0.975 & 0.990 (w/o) 0.904,0.979 & 0.976	O‐MAR application reduced the inter‐reader variability of area measurements
Hansen et al.^52^	O‐MAR	Clinical (H&N); (n = 11)	Dental implants	CT_art_ & CT_cor_	GTV‐Tumor GTV‐lymph node Parotid	Volume (cm^3^) [Mean ± STD] DSC of contours [Mean ± STD]	16.1 ± 10.2 & 19.7 ± 13.9 12.5 ± 8.9 & 12.9 ± 9.5 20.0 ± 7.7 & 21.3 ± 7.6 0.60 ± 0.24 & 0.61 ± 0.20 0.75 ± 0.11 & 0.75 ± 0.09 0.70 ± 0.17 & 0.75 ± 0.17	O‐MAR improved the volume delineation of structures in a constant way
Hagen et al.^53^	iMAR	Clinical (H&N &hip); (n = 2)	Dental implants and bilateral hip implant	CT_art_ & CT_cor_ CT_ref_, CT_art_ & CT_cor_ (CT_art_ vs CT_ref_) & (CT_cor_ vs CT_ref_)	GTV‐tongue GTV‐prostate	Volume (cm^3^) [Mean ± STD] DSC [Mean ± STD]	34, 28 ± 6 & 30 ± 7 87 ± 44 & 75 ± 22 0.83 ± 0.06 & 0.86 ± 0.06 0.68 ± 0.15 & 0.78 ± 0.07	iMAR improved the accuracy of the organ contouring
Axente et al.^35^	iMAR	Clinical (hip, H&N, spine, knee & breast); (n = 8)	Metal implants & metal prosthesis	CT_art_ & CT_cor_	Selected structures	Visual conspicuity score [Median]	3 of 5 & 4 of 5	iMAR improved the accuracy and efficiency of contouring
Kovacs et al.^54^	iMAR	Clinical (H&N, spine & hip); (n = 1)	Metal implants	CT_art_, CT_cor_ & CT_ref_	H&N Spine Hip	HD (mm) [Median] DSC of contours [Mean]	10.7, 5.1 & 2.9 18.2, 18.6 & 2.2 7.7, 3.5 & 2.0 0.75, 0.87 & 0.91 0.57 & 0.74 & 0.88 0.5 & 0.87 & 0.95	iMAR improved the contouring accuracy
Shiraishi et al.^55^	SEMAR	Clinical (Pelvis); (n = 40)	Iodine seeds with Ti & Ag	(CT_art_ vs CT_ref_) & (CT_cor_ vs CT_ref_)	Prostate gland	TPF [Mean ± STD]	0.982 ± 0.0159 & 0.992 ± 0.0103	SEMAR improved the accuracy of seed localization in brachytherapy

H&N Head and neck, Ti Titanium, Ag Silver, CT_art_ CT scans with artifacts, CT_cor_ Corrected CT scans, CT_ref_ Reference CT scans, vs versus, ICC Inter coefficient correlation, DSC Dice similarity coefficient, HD Hausdorff distance, TPF True‐positive fraction, (w/o) without outliers and STD Standard deviation.

In another study by Andersson et al., visual grading was used for the evaluation of anatomical delineation.^50^ A comparison between CT_art_ and either O‐MAR or iMAR‐corrected CT_cor_ was performed. Visual grading resulted in significantly (*P < 0.001*) higher scores on both CT_cor_. However, new induced artifacts were also identified on CT_cor_. Kohan et al. used clinical H&N CT scans with dental implants for their O‐MAR evaluation.^51^ Reviewers with different experience levels in contouring performed area measurements of selected structures on CT_art_, CT_cor_ after O‐MAR application, and CT_ref_. The intraclass correlation coefficient (ICC) was calculated to assess inter‐reader variability. The highest ICC value of 1 indicates the lowest inter‐reader variability, while ICC value of 0 indicates the highest inter‐reader variability. For all reviewers, the ICCs for CT_art_, CT_cor_ after O‐MAR application, and CT_ref_ were 0.884, 0.971, and 0.989, respectively, without outliers; and 0.903, 0.948, and 0.985, respectively, with outliers. For the experienced readers, the ICC for CT_art_, CT_cor_ after O‐MAR application, and CT_ref_ were 0.934, 0.975, and 0.990, respectively, without outliers; and 0.904, 0.979, and 0.976, respectively, with outliers. Application of O‐MAR improved the ICC values and brought them closer to the reference ones. For this reason, CT_cor_ after O‐MAR application reduced the inter‐reader variability during contouring. Another study by Hansen et al. also concluded that the application of O‐MAR on clinical H&N CT_art_ increased the organ delineation and contouring accuracy.^52^ The authors also measured and compared the gross tumor volume (GTV) and parotid volume on CT_cor_ after O‐MAR application and on CT_art_. Removal of streak artifacts increased the signals from the artifact corrupted areas, thus depicting a consistently larger contoured GTV (mean 22%, *P < 0.06*) and parotid volume (mean 7%, *P = 0.05*). However, the authors noted that to determine the actual volumes of the delineated structures the measured volumes should be compared with the reference volumes. A similar finding was reported by Hagen et al.; they found that contouring on CT_cor_ after iMAR application would increase the mean GTV tongue in H&N cases.^53^ The GTV tongue increased (*P = 0.267*) from 28 ± 6 cm^3^ (mean ± STD) on CT_art_ to 30 ± 7 cm^3^ (mean ± STD) on CT_cor_. However, the mean volume of the parotid as an OAR was reduced in this study. Moreover, the authors evaluated the size of the prostate GTVs on bilateral implanted pelvis CT scans, and they were reduced (*P = 0.168*) from 87 ± 44 cm^3^ (mean ± STD) on CT_art_ to 75 ± 22 cm^3^ (mean ± STD) on CT_cor_ after iMAR application. For the OARs on CT_cor_ after iMAR application in the pelvis case, the mean volume for rectum and bladder was reduced and increased, respectively. The DSC of the contours with respect to the reference increased more for the CT_cor_ after iMAR application than for the CT_art_. Both the GTV of the tongue and the prostate on CT_cor_ after iMAR application were underestimated in comparison with the predefined reference. Nevertheless, it improved the confidence in contouring, as indicated by higher DSC values. Axente et al. assessed the image quality and visual conspicuity of CT_art_ and CT_cor_ after iMAR application.^35^ Different types of clinical images were used, such as hip cases with unilateral or bilateral metal implants, H&N cases with dental fillings, a spine with metal implants, a knee with prosthesis, and a breast with expander. The median score for the image quality and visual conspicuity of CT_art_ and CT_cor_ after iMAR application increased from 3 to 4 of 5. During the image quality assessment, new secondary artifacts were identified on CT_cor_. Another study investigated iMAR for its anatomical delineation accuracy.^54^ For this study, clinical CT scans (H&N, spine, and hip) with metal implants were used. Maximum Hausdorff (HD) distance and DSC were calculated to quantify the anatomical delineation accuracy. High HD distance indicates large difference between two contours while short HD distance indicates small variation. Contouring was performed on CT_art_ and CT_cor_ after iMAR application and the maximum HD distance with respect to contours on CT_ref_ was 10.7 mm and 5.1 mm on dental scans, 18.2 mm and 18.6 mm on spine scans, and 7.7 mm and 3.5 mm on hip scans, for CT_art_ and CT_cor_, respectively. In dental and hip scans, the maximum HD distance on CT_art_ had the largest values and differed significantly (*P* < 0.05) from CT_ref_. Furthermore, the calculated DSC values for contours on CT_art_ and CT_cor_ after iMAR application were 0.75 and 0.87 on dental, 0.57 and 0.74 on spine, and 0.5 and 0.87 on hip, respectively. The higher DSC values on CT_cor_ after iMAR application indicate that there is less variability in contouring and a higher accuracy.

The SEMAR algorithm was evaluated by Shiraishi et al. to quantify its ability to improve the detection accuracy of implanted iodine seeds which contain Ti and silver (Ag) in brachytherapy.^55^ To identify seeds on both CT_art_ and CT_cor_ after SEMAR application an automatic seed finder was used, and the results were compared with reference positions. The mean true‐positive fraction (TPF) was calculated, and it had significantly higher values (P < 0.05) for CT_cor_ after SEMAR application (0.992 ± 0.0103, [mean ± STD]) than for CT_art_ (0.982 ± 0.0159, [mean ± STD]). Thus, the application of SEMAR on CT_art_ improved implanted seed detection.

Overall, CT_cor_ after application of the above‐mentioned commercial MAR algorithms improved the anatomical conspicuity and contouring accuracy. On the other hand, new artifacts induced by the MAR algorithms appeared on resulting CT_cor_ and it is clear that the external factors such as physician’s knowledge and experience considerably influence these results.

#### 3.A.3 Dose‐based evaluations and corrections

##### Dosimetric impact of commercial MAR algorithms

The commercial MAR algorithms were also evaluated to assess their ability to improve the dose calculation accuracy in RT. Table 3 provides a summary of these research studies. O‐MAR was evaluated by Kwon et al. in clinical and phantom studies.^41^ Clinical H&N CT scans with dental implants were acquired in open‐mouth and closed‐mouth positions. In the close‐mouth case, the nasopharynx, the parotid, and the submandibular salivary gland were contoured as targets and dose calculation was performed on CT_art_ and CT_cor_ after O‐MAR application. A nonsignificant (*P* > 0.05) mean gamma passing rate (%GP) of 99.4 ± 0.5% (mean ± STD) was reported between them using a 1%/1 mm gamma criterion. However, for targets such as the tongue and the tonsil, a large discrepancy in %GP was found between CT_art_ and CT_cor_ after O‐MAR application under the same criteria in the open‐mouth scans. A virtual build‐up region was created in front of the oral cavity due to the metal artifacts on CT_art_ which resulted in this dose discrepancy. Furthermore, the phantom studies revealed that the calculated dose on CT_cor_ after O‐MAR application was closer to the delivered dose (measured with films) than the calculated dose on CT_art_. Also Huang et al. studied the application of O‐MAR to improve dose calculation using phantoms.^56^ A custom‐made geometric phantom with inserts of Ti or Cerrobend, and two anthropomorphic phantoms, one with spinal implants and another one with dental fillings, were used. In the geometric phantom with Ti insert, the dose errors between calculated and measured dose with a 2%/2 mm gamma criterion were 15% for CT _art_ and 11.1% for CT_cor_ after O‐MAR application in the region which was under the metal implant. Furthermore, in the anthropomorphic phantoms’ evaluation, the O‐MAR application improved the dose calculation accuracy in the dental filling case while it had little impact for the spinal implant case. Similar findings were reported by Sillanpaa et al. for clinical H&N CT scans.^49^

**Table 3:** Summary of the studies which investigated the dosimetric impact of commercial MAR algorithms in RT.

Author	MAR Algorithm	Images; No. of sample (n)	Metal	Type of radiation, Energy, and (Mode of therapy)	Dose calculation algorithm (TPS); [Dose measurement]	CT Scans	Calculation and Measurement	Results	Comments
Kwon et al.^41^	O‐MAR	Phantom Clinical (H&N); [open mouth (n = 3) & closed mouth (n = 8)]	Al, Ti, Cr, & Zr Dental implants	Photon, 6 MV (VMAT)	AAA, (Eclipse™, Varian) [Gafchromic®, EBT2]	CT_art_ & CT_cor_	Discrepancy in calculated dose (%) (3%/3mm) %GP (3%/3 mm) Mean %GP, on closed mouth (1%/1mm) %GP, on open mouth (1%/ 1mm)	95.3% (Ti) & 91.6% (Cr) 83.60% & 86.68% (Ti) 79.70% & 86.30% (Cr) 99.40 ± 0.5% 91.1%, 94.8% & 96.6%	Dose calculations after O‐MAR application are closer to the measured dose
Huang et al.^56^	O‐MAR	Geometric phantom Anthropomorphic phantom	Ti & Cerrobend Ti & Amalgam	Photon, 6 MV (IMRT)	CCC (Pinnacle (Philips) [Gafchromic® EBT2 & ion chamber]	CT_art_ & CT_cor_ CT_cor_ vs measurement CT_art_ vs CT_cor_	Dose calculation error (%) (2%/2mm) Dose calculation error (%) (2%/2mm)	15% & 11.1% < 1% (Ti) 6.7% & 1.8% (Amalgam)	O‐MAR had little dosimetric impact on Ti, and large impact on amalgam.
Sillanpaa et al.^49^	O‐MAR	Clinical (H&N); (n = 28)	Dental implants	Photons, 6 MV (IMRT & VMAT)	AAA, algorithm (Eclipse™, Varian)	(CT_art_ ‐ CT_cor_)/ ( CT_cor_)	Percent change Mean dose—PTV PTV V_95%_	‐0.3% ‐0.2%	O‐MAR algorithm did have an insignificant dosimetric effect
Shen et al.^57^	O‐MAR	Phantom Clinical (Spine); (n = 5)	Ti Spinal prosthesis	Photon, 6MV (IMRT) Photon, 6MV (IMRT)	CCC (Pinnacle, Philips) PB (iPlan, BrainLAB Inc.)	CT_art_ & CT_cor_	%GP (2%/2 mm) (1%/1 mm) %GP (2%/2 mm) (1%/1 mm)	> 99.98% > 99.96% 99.33% & 100% 96.96% & 99.93%	O‐MAR did not affect the dose calculation accuracy for SBRT planning in patients with spinal implants.
Akdeniz et al.^59^	O‐MAR	Phantom	SS & Ti	Photons, 6 MV	AAA and AXB (Eclipse™, Varian) [Gafchromic™ EBT3]	(CT_art_ vs measurement) & (CT_cor_ vs measurement)	Dose differences at upper SS surface (%) in AXB in AAA Dose differences at lower Ti surface (%) in AAA	23.1% & 16.9% 18.2% & 12.7% ‐13.30% & −11.50%	Dose calculation after application of O‐MAR with AXB shows closer dose agreement with dose measurements
Jia et al.^62^	O‐MAR	Phantom	SS	Proton	(Eclipse™, Varian)	CT_cor_ & CT_art_	Relative dose discrepancy	20% at 2 mm & 80% at 4 mm	O‐MAR improved the accuracy of dose distribution
Righetto et al.^63^	O‐MAR	Phantom	Ti	Proton	MC	(CT_art_ vs reference) & (CT_cor_ vs reference)	ΔWET (cm)	0.16 cm & 0.57 cm	Application of O‐MAR overestimated the ΔWET
Andersson et al.^50^	O‐MAR & iMAR	Phantom Clinical (H&N); (n = 30)	Dental filling Neck implant Dental implants	Proton, from 60 MeV to 226 MeV	Proton pencil beam (Eclipse™, Varian)	CT_art_ & CT_cor_ CT_cor_ CT_art_ & CT_cor_	ΔWET (mm) O‐MAR iMAR Residual ΔWET O‐MAR iMAR %GP (whole image)	‐17.0 mm & −4.3 mm ‐16.1 mm & −2.3 mm ‐2.3mm ‐1.5 mm ~ 97%	O‐MAR and iMAR improved the dose distribution. However, case‐by‐case evaluations before applying MAR is important
Bär et al.^42^	iMAR	Phantom Clinical (H&N); (n = 7) Clinical (Pelvis); (n = 1)	Al, Ti & SS Dental implant Hip bilateral implant	Photon, 6MV (IMRT)	CCC (RaySearch)	(CT_cor_ & CT_art_) vs CT_ref_ CT_art_ & CT_cor_	%GP (1%/1mm) Dose calculation error (%)	85.70% & 62.10% up to ±5%	iMAR improved the dose calculation accuracy
Maerz et al.^43^	iMAR	Phantom	Dental implants	Photon (IMRT & VMAT)	PB and CC (Oncentra External Beam v4.3)	CT_art_ & CT_cor_	%GP (3 % / 3 mm)	90.6 % & 96.2 %	iMAR tends to improve the dose calculation accuracy
Axente et al.^35^	iMAR	Phantom	Al & SS	Photon, 6MV & 15MV (VMAT, 3D CRT) Proton, 195 MeV	AXB (Eclipse™, Varian) PBCS (Eclipse™, Varian)	CT_art_ & CT_cor_	%GP for 6MV (2%/2mm) (2%/1mm) %GP, for 15MV (2%/2mm) (2%/1mm) %GP (2%/2mm) (2%/1mm)	97% & 99.4% 92.1% & 98.1% 97.5% & 99.6% 92.9% & 98.3% 88.6% & 91.9% 78.7% & 91%	iMAR improved the dose calculation accuracy in proton and photon beams
Huang & Kohli^45^	Smart MAR	Clinical (H&N); (n = 15) Clinical (Pelvis); (n = 10)	Dental filling Hip prosthesis	Photons, 6MV IMRT IMRT & VMAT	AAA (Eclipse™, Varian)	CT_cor_ & CT_art_	Mean ± STD difference (%) CI D_99%_ V_100%_ CI D_99%_ V_100%_	‐0.3 ± 0.9% ‐0.1 ± 0.1% ‐0.1% ± 0.5% ‐8.8 ± 11.4% ‐0.1 ± 0.4% ‐8.8% 12.1%	Dosimetric differences between CT_cor_ after Smart MAR and CT_art_ were small
Inal & Sarpün^46^	Smart MAR	Phantom	Leads	Photons, 6MV (IMRT)	CS (Elekta XiO) [Matrix arrays]	(CT_cor_ vs measurement) & (CT_art_ vs measurement)	Mean %GP difference (3% /3 mm) 5 fields 7 fields 9 fields	94.98% & 96.11% 94.72% & 95.90% 91.34% & 92.83%	Smart MAR increased the gamma passing rate
Guilfoile et al.^44^	Smart MAR	Phantom	Hip prosthesis	Photon, 6MV, 10MV & 15 MV	Pinnacle (Philips) [ion chamber]	(CT_cor_ vs measurement) & (CT_art_ vs measurement)	Dose difference (%)	Refer to fig. 6 in Ref. [44]	Smart MAR improved the dose calculation accuracy in treatment planning
Murazaki et al.^47^	SEMAR	Phantom	Unilateral & bilateral metal	Photons, 10MV (one filed irradiation, opposite portal irradiation, four field irradiation, & VMAT)	AAA and AXB (Eclipse™, Varian)	CT_ref_ vs CT_art_ CT_ref_ vs CT_cor_	%GP (1%/0 mm) in AXB with opposite portal irradiation	89.89% 95.03%	Application of SEMAR improved the accuracy of dosimetry in treatment planning
Miki et al.^48^	SEMAR	Phantom Clinical (H&N); (n = 2)	Metal screws Dental implants	Carbon ion	N/A	CT_art_ & CT_cor_	Mean %GP (3%/ 3mm) (2%/2mm) PTV‐D95% %GP (3%/3mm) Clinical 1 Clinical 2 PTV‐D95% Clinical 1 Clinical 2	89.1% 79% 82.4% & 95.4% 99.9% 92.7% 92.2% & 92.5% 90.9% & 95.7%	Application of SEMAR improved the dose distribution
Shiraishi et al.^55^	SEMAR	Clinical (pelvis); (n = 40)	Iodine capsules with Ti & Ag	Brachytherapy	VariSeed 8.0 (Varian Medical Systems)	(CT_art_ vs CT_ref_) & (CT_cor_ vs CT_ref_)	Median difference D_90%_ V_100_% V_150_%	0.78% & 0.28% 0.24% & 0.06% 1.70% & 0.58%	Prostate postimplant CT_cor_ after SEMAR improved the postimplant dosimetric parameters

H&N Head and neck, Ti Titanium, SS Stainless Steel, Ag Silver, Cr Chromium, Zr Zirconia, IMRT Intensity‐Modulated Radiation Therapy, VMAT Volumetric‐Modulated Arch Therapy, CCC Convolution Collapsed Cone, PB Pencil Beam, AAA Anisotropic Analytical Algorithm, AXB Acuros®XB, CS Convolution Superposition, MC Monte‐Carlo, CT_art_ CT scans with artifacts, CT_cor_ Corrected CT scans, CT_ref_ Reference CT scans, vs versus, %GP gamma passing rate, ΔWET Deviation in Water Equivalent Thickness, PTV Planning Target Volume.

The dosimetric impact of O‐MAR on the spine for stereotactic body radiation therapy (SBRT) was evaluated by Shen et al. using a phantom and clinical CT scans.^57^ SBRT can deliver high ablative radiation dose to the target, while sparing OARs.^58^ A CT electron density phantom (Gammex, Model RMI 465, Middleton, WI, USA) with a Ti implant was used in this study. A similar calculated planar dose distribution was observed on CT_art_ and CT_cor_ after O‐MAR application and %GP was larger than 99.98% for a 2%/2 mm gamma criterion and 99.96% for a 1%/1 mm gamma criterion. The evaluation of the clinical CT scans revealed similar findings. Thus, the study concluded that O‐MAR does not significantly affect the dose calculation accuracy and can, therefore, be safely used for SBRT treatment planning. In another recent study, Akdeniz et al. evaluated the dosimetric effects of metal implants in small‐field RT using a custom‐made slab phantom (PTW, Freiburg, Germany).^59^ The study revealed that small differences in dose were observed between the calculated and measured dose on both CT_art_ and CT_cor_ after O‐MAR application. The authors found that the type of dose calculation algorithms available on the TPS also influences the dose differences. The Anisotropic Analytical Algorithm (AAA)^60^ and Acuros External Beam (AXB) algorithm^61^ available in the Eclipse™ TPS (Varian Medical Systems, Palo Alto, CA) were evaluated. On CT_cor_ after O‐MAR application the use of the AXB algorithm better reduced the dose differences between the calculated and measured dose compared to the AAA algorithm.

Jia et al. studied the dosimetric impact of O‐MAR on proton therapy treatment planning using a solid water phantom with a SS crew inserted.^62^ The discrepancies of relative depth dose distribution were calculated on CT_cor_ after O‐MAR application and CT_art_, and they were 2 mm at 20% relative dose and 4 mm at 80% relative dose, respectively. The O‐MAR and iMAR algorithms were evaluated for proton therapy in phantom and clinical studies by Andersson et al.^50^ A head phantom (CIRS, Model 731‐HN, Norfolk, VA, USA) was CT scanned with and without a removable dental filling and a neck implant. Deviation in water equivalent thickness (ΔWET) was calculated to find the proton range errors on CT_ref_, CT_art_, and CT_cor_. In case of the dental filling, along a dark streak, ΔWET was improved from −17.0 mm to −4.3 mm by O‐MAR, and from −16.1 mm to −2.3 mm by iMAR. For other directions, ΔWET increased or remained unchanged on CT_cor_. Generally, ΔWET was reduced in case of the neck implant; however, residual deviations up to −2.3 mm with O‐MAR and up to −1.5 mm with iMAR remained. In the clinical H&N study with dental implants, planned dose distributions to a neck node were calculated on CT_art_ and CT_cor_, and minor differences were observed. On the other hand, in a phantom (Gammex Inc., Middleton, WI, USA) study with a Ti implant, Righetto et al. reported that the calculated proton range using WET was overestimated more on the CT_cor_ after O‐MAR application than on both CT_art_ and CT_ref_.^63^ The reference was obtained using stopping power values from the data which were published by NIST (Gaithersburg, MD, USA). As a result, ΔWET on CT_cor_ after O‐MAR application and CT_art_ were 0.57 cm and 0.16 cm, respectively, when compared with the reference value.

Bär et al. focused their work on the iMAR algorithm using CT scans from the Gammex 467 phantom with and without the metal inserts, and the clinical CT scans of H&N and a hip with implants.^42^ In the phantom, a target was contoured in the center between two implanted metals, and dose calculations were performed on CT_ref_, CT_art_, and CT_cor_ after iMAR application. A %GP for a 1%/1 mm gamma criterion of 62.1% was found between the CT_ref_ and CT_art_ while CT_cor_ improved it up to 85.7%. In the clinical CT scans, dose differences up to ±5% have been shown when comparing the plans which were calculated on CT_cor_ and CT_art_. The application of iMAR reduced the dose errors and, therefore, the authors recommended to use this method for RT applications. A similar finding was reported by another cylindrical phantom study with dental implants. The dose distributions were calculated on CT_art_ and CT_cor_ and it was shown that the application of iMAR improved the %GP from 90.6% to 96.2% for a 3%/3 mm gamma criterion.^43^ In the CIRS phantom study,^35^ the calculated dose distribution on CT_cor_ after iMAR application was closer to the CT_ref_ dose distribution than the dose distribution calculated on CT_art_. In addition to dose distributions, the %GP of photons (6 MV and 15 MV) and protons (195 MeV) were derived from the calculated dose distributions on CT_art_ and CT_cor_. For the 6MV photon beam, iMAR increased the photon %GP for a 2%/2 mm gamma criterion from 97% to 99.4% and for the 15 MV photons similar results were shown. For the proton beam, iMAR increased the %GP from 88.6% to 91.9% under the same criteria.

Smart MAR was evaluated for dose calculation accuracy in RT by Huang and Kohli using clinical CT scans of H&N with dental fillings and of a pelvis with a metal prosthesis.^45^ The average conformity index (CI), D_99%_, and V_100%_ were calculated on CT_cor_ after Smart MAR application and CT_art_ and then compared. The average percentage (mean ± STD) differences in CI, D_99%_, and V_100%_ on H&N CT scans were −0.3% ± 0.9%, −0.1% ± 0.1%, and −0.1% ± 0.5%, respectively. For the CT scans of the pelvis, they were (mean ± STD) −8.8% ± 11.4%, −0.1% ± 0.4%, and −8.8% ± 12.1%, respectively. Also, this study found that the calculated dose differences between the CT_cor_ and CT_art_ were not significant. In another study, Inal and Sarpün evaluated Smart MAR for dose calculation accuracy in 12 different intensity‐modulated radiation therapy (IMRT)^64^ plans with 5‐, 7‐, and 9‐field beam arrangements and segment numbers.^46^ IMRT is an inverse planning‐based treatment delivery method which is optimized until the target volume and normal tissue reached the prescribed dose. A custom‐made metal‐containing phantom was CT scanned. For the five, seven, and nine fields, the calculated dose distributions on CT_art_ and CT_cor_ after Smart MAR application were compared with the measured dose. The %GP for a 3%/3 mm gamma criterion of the above‐mentioned dose distribution were 94.98% and 96.11% (for five fields), 94.72% and 95.90% (for seven fields), and 91.34% and 92.83% (for nine fields), respectively. The improvement of %GP on CT_cor_ shows that Smart MAR increased the dose calculation accuracy. A similar finding was reported by Guilfoile et al. using a custom‐made hip phantom with bilateral hip prostheses.^44^

Murazaki et al. evaluated the SEMAR algorithm for dose calculation accuracy using a standard electron density phantom (CIRS, Model 062A, Norfolk, VA, USA) with metal inserts.^47^ Several different treatment plans were prepared, using forward planning with one field, with two opposite fields, and four fields; and with volumetric‐modulated arc therapy (VMAT).^65^ The AXB and AAA algorithms were used to calculate the dose distribution on CT_art_ and CT_cor_ after SEMAR application. In AXB for the two opposite fields, SEMAR increased the %GP for a 1%/0 mm criterion from 89.89% to 95.03 %, and similar results were observed also in AAA as well as for the other forward planning methods under the same criteria. This implies that the calculated dose distribution was improved with the use of SEMAR. Miki et al. studied the dosimetric impact of the SEMAR algorithm in carbon ion therapy using phantom and clinical CT scans.^48^ An anthropomorphic head phantom was scanned with and without metal inserts and then SEMAR was applied to create CT_cor_ from the CT_art_. A planning target volume (PTV)^66^ was placed on the dark streak band and PTV‐D_95%_ was measured. The calculated PTV‐D_95%_ on clinical H&N scans with dental implants and with tumors near the spinal cord as the treatment target showed a higher PTV‐D_95%_ (from 82.4% to 95.4%) for CT_cor_ than for CT_art_. To investigate brachytherapy applications, Shiraishi et al. used clinical pelvis CT scans with implanted iodine seeds (which contain Ti and Ag).^55^ SEMAR was applied to generate CT_cor_ from CT_art_, and then D_90%_, V_100%,_ and V_150%_ were calculated on those CT scans and compared with CT_ref_. The differences in dosimetric calculations were significantly smaller (*P < 0.05*) between CT_cor_ and CT_ref_ than between CT_art_ and CT_ref_.

In general, the CT_cor_ after application of a commercial MAR algorithm improves the dose calculation accuracy in RT. This improvement is indicated by increment of %GP, reduction of errors in dose calculation compared to CT_art_, and minor differences from the measured dose using films and/or ion chambers or calculated dose on CT_ref_. Furthermore, improvements in the WET calculation were also reported after artifact correction by commercial MAR algorithms in proton therapy. However, the amount of improvement in calculated dose depends on the utilized dose calculation algorithm and on radiation therapy technique. Studies reported that the AXB algorithm increases the dose calculation accuracy more than the AAA. Moreover, application of VMAT is preferable to improve the accuracy in calculated dose compared to IMRT.

##### Dosimetric impacts of density correction methods

The density correction methods which are available in the TPSs can also be used to reduce the metal artifacts on CT scans for RT applications, see Table 4. Maerz et al. used a dental implant cylindrical phantom to evaluate dose calculation accuracy after application of a density correction method.^38^ IMRT and VMAT plans were calculated on CT_art_ and CT_cor_ after density correction and then compared with dose measurements using films. Findings revealed that the accuracy of dose calculation was higher (*P = 0.015*) on CT_cor_ than on CT_art_ for both IMRT and VMAT plans. Moreover, the VMAT plan increased the %GP for a 3%/3mm gamma criterion in comparison with the IMRT plan on both CT_art_ and CT_cor_. On the other hand, Acquah et al. used a phantom (CIRS, Model 002LFC, Norfolk, VA, USA) to compare the calculated dose on CT_cor_ after density correction with the measured dose using ion chambers.^67^ The authors found that treatment planning on CT_cor_ gave a 16% higher average dose discrepancy. The dose discrepancy between the calculated dose on CT_art_ and the measured one was not mentioned in this study. This study suggested that caution should be taken while planning on CT_cor_ after density correction. Parenica et al. evaluated the impact of a density correction method on a VMAT plan using CT scans of a custom‐made phantom and on clinical pelvis CT scans with hip prostheses.^68^ Densities of the prostheses and surrounding tissue which contain metal artifacts were overridden with the appropriate corrected density. Dose calculation algorithms with collapsed cone convolution superposition (CCCS)^69^ available on Pinnacle (Philips, Fitchburg, Wisconsin) and a Monte Carlo^70^‐based algorithm available in Monaco (ELEKTA, Stockholm, Sweden) TPS were used. The calculated dose on CT_art_ and CT_cor_ after density override was compared with the respective measured dose using a thermoluminescence dosimeter (TLD). In the phantom, the dose errors were 9.2% and 4.4% for Pinnacle and 3.6% and 0.2% for Monaco, respectively. For the clinical pelvis scans, the prostate was contoured as the target. Subsequently, a comparison of the calculated average D_95%_ without and with density correction was 99.3% and 82.7% for Pinnacle and 99.0% and 90.6% for Monaco, respectively. The reduced D_95%_ on CT_cor_ resulted from a reduced PTV. The same authors in another study^71^ found similar results for density correction method on data of a phantom with Al, Zn, and SS inserts, and clinical H&N CT scans with dental implants.

**Table 4:** Summary of the studies which investigated the dosimetric impact of density correction in RT.

Author	Images; No. of sample (n)	Metal	Beam, Energy, and (Mode of therapy)	Dose calculation algorithm (TPS); [Dose measurement]	CT Scans	Calculation and Measurement	Results	Comments
Maerz et al.^38^	Phantom	Dental implants	Photon, 6 MV (VMAT & IMRT)	PB and CC (Oncentra External Beam)	CT_art_ & CT_cor_	%GP IMRT VMAT	0.954% & 0.980% 0.983% & 0.990%	Density correction increased the dose calculation accuracy
Acquah et al.^67^	Phantom	Spine implant	Photons, 6 MV & 15MV (3D‐CRT)	CC & PB (Oncentra External Beam)	CT_cor_ vs CT_art_	Mean dose discrepancy (%)	16%	Density correction resulted in higher dose discrepancy
Parenica et al.^68^	Phantom Clinical (Pelvis); (n = 6)	Ti Hip prosthesis	Photons, 6 MV (VMAT)	CCC (Pinnacle, Philips) & MC (Monaco) [Ion chamber]	(CT_art_ & CT_cor_) vs Measured CT_art_ & CT_cor_	Dose errors (%) Pinnacle Monaco Average D_95%_ Pinnacle Monaco	9.2% & 4.4 % 3.6% & 0.2% 99.3% & 82.7% 99.0% & 90.6%	Density override can be used to optimize the dose calculation planning with MC
Parenica et al.^71^	Phantom Clinical (H&N); (n = 9)	AL, Zn & SS Dental implants	Photons, 6 MV (VMAT)	CCC (Pinnacle, Philips) & MC (Monaco) [TLD]	(CT_art_ & CT_cor_) vs Measured CT_art_ & CT_cor_	Dose errors (%) for Al Pinnacle Monaco for Zn Pinnacle Monaco for SS Pinnacle Monaco D_95%_	4% & 4% 3.9% & 1.7% 8.6% and 7.1% 9.8% and 2.3% 8.8% and 5.8% 9.5% and 3.0% Refer to Table 5 in Ref. [71]	The density override reduced the potential risk of compromising the dose to the target and healthy tissues

Ti Titanium, SS Stainless Steel, Ag Silver, Zn Zinc, CRT Conformal Radiation Therapy, IMRT Intensity‐Modulated Radiation Therapy, VMAT Volumetric‐Modulated Arch Therapy, CCC Convolution Collapsed Cone, PB Pencil Beam, MC Monte‐Carlo, CT_art_ CT scans with artifacts, CT_cor_ Corrected CT scans, CT_ref_ Reference CT scans, vs versus, %GP gamma passing rate.

### 3.B Research MAR methods

#### 3.B.1 Traditional MAR algorithms

Several research MAR algorithms which utilize traditional image processing methods have been proposed and/or evaluated for the reduction in metal artifacts on CT scans for RT applications. These algorithms rely on several different principles. Some approaches are based on image inpainting or sinogram inpainting^72^, ^73^, ^74^, ^75^; some others require the acquisition of additional tilted CT scans,^76^ propose novel image acquisition, and reconstruction methods^77^, ^78^; or require the use of magnetic resonance imaging (MRI)^79^, ^80^ or megavoltage CT (MVCT).^81^, ^82^

**Fig. 4:**
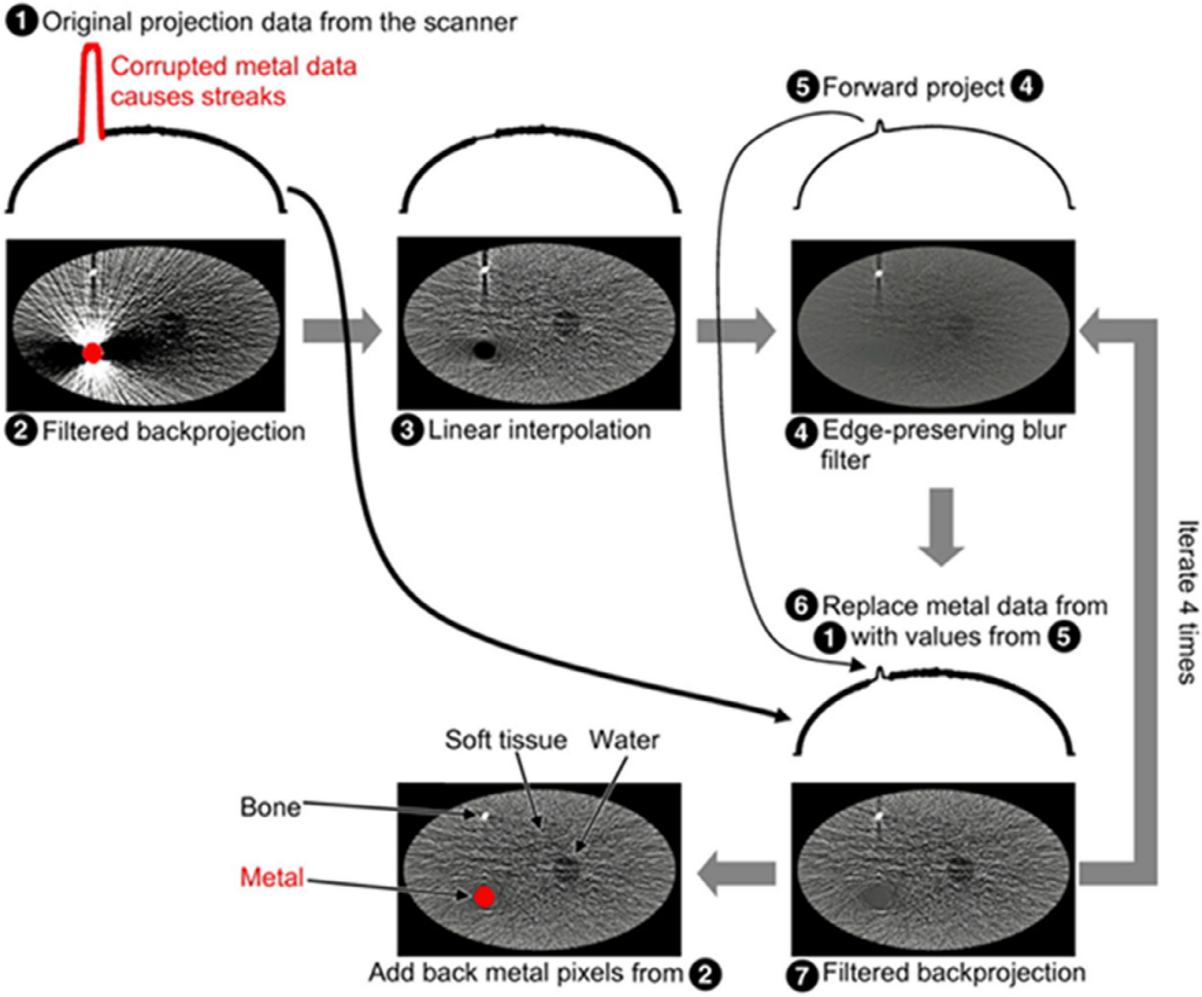
Working principle of the MDT MAR algorithm. A metal segmented CT slice (image 2) undergoes linear interpolation (image 3) and edge preserving filtration (image 4). Then, the filtered image is forward projected (number 5) and used to replace the metal data on the original projection (number 1). The resulting projection (number 6) is filtered back projected and undergoes edge preserving filtering. This process is iterated four times and results in a metal artifact reduced CT slice (image 7). Finally, the segmented metal is added to create the artifact‐corrected CT slice. (Reproduced, with permission, from Boas F E, Fleischmann D. Evaluation of Two Iterative Techniques for Reducing Metal Artifacts in Computed Tomography. Radiology 2011; 259:894‐902. The Radiological Society of North America (^©^RSNA)).

For the metal deletion technique (MDT)^75^ (Fig. 4) which uses sinogram inpainting iteratively, initially pixels which contain metal data are segmented from CT_art_ (Fig. 4, image 2). Then, linear interpolation (LI)^83^ and edge‐preserving blur filters (Fig. 4, image 4) are applied on this CT scan to calculate the missing pixel values and to reduce the noise, respectively. Subsequently, the linearly interpolated and noise‐reduced image is forward projected to create an initial sinogram (Fig. 4, number 5). This sinogram is used iteratively (four iterations in total) to replace the pixels which contain metal artifacts in the original sinogram. On each iteration, rays that pass through the metal are replaced with the value from the previous iteration (Fig. 4, number 6). This procedure results in a corrected sinogram. Finally, the filtered back‐projection of the corrected sinogram (Fig. 4, image 7) with added metal data produces the CT_cor_.

An MRI‐based MAR algorithm was proposed by Park et al.^79^ The proposed method reduces the metal artifacts by mapping the HU values from a nearby artifact‐free CT slice using a coregistered MRI scan. Initially, the CT_art_ slice and adjacent artifact‐free slice are manually identified and then registered with their corresponding MRI slice. Based on the intensity values of pixels on the paired MRI slice and HU values from the artifact‐free CT slice, HU values of pixels on CT_art_ slice are calculated. Nielsen et al. evaluated their proposed MR‐based MAR algorithm (kerMAR)^80^ to reduce the metal artifacts.^84^ kerMAR requires aligned CT and MRI scans of the same anatomy. It uses a Bayesian modelling^85^ approach to compute the corrected HU values of a corrupted CT slice from the corresponding coregistered MRI slice.

Liugang et al. proposed a MAR method which makes use of megavoltage computed tomography (MVCBCT) and kilovoltage computed tomography (kVCT) scans for MAR.^81^ Initially, the metal part in the MVCBCT scan is segmented and forward projected to obtain the metal trace. Then, a prior image is obtained by combining both scans through the fusion method proposed by Wang *et al*.^86^ and this image is forward projected to obtain missing data to replace the metal trace on the kVCT scan. Finally, the kVCT scan with reduced metal artifacts is created through filtered back projection. In a similar study by Jeon et al., a hybrid sinogram‐based MAR method was proposed for helical tomotherapy.^82^ It also requires a kVCT scan and a MVCT scan of the same anatomy. During the MAR application, it replaces the metal affected signals of the forward‐projected kV sinogram with the corresponding forward‐projected MV sinogram. Finally, the application of filtered back projection on the corrected kVCT sinogram produces a kVCT scan with corrected artifacts.

A study by Kim et al. proposed to acquire an additional tilted CT scan in which less metal artifacts are present.^76^ First, an artifact map is generated from the denoised initial CT scan (CT_art_) and the additional tilted CT scan. Then, utilizing the SSIM metric, correlation maps are generated from the CT_art_ and the additional CT scan in comparison with the artifact map. A minimum value in the correlation map represents smaller amounts of metal artifacts and, thus, the CT_cor_ is reconstructed using the minimum correlation values.

A new CT reconstruction algorithm, augmented likelihood image reconstruction (ALIR)^78^ was investigated for MAR for RT applications. It utilizes an iterative scheme and prior information of the metal implant such as shape and attenuation coefficient. A new sinogram is created by masking the metal on the initial sinogram. Using this new sinogram an image is iteratively reconstructed using log‐likelihood function^87^ and prior information. During each iteration, a bilateral filter is applied on the reconstructed image to reduce the remaining metal artifacts and then a forward projection of this images is used to calculate new projection values, which are combined with the initial projection values thereafter. Finally, the CT scan is reconstructed with reduced metal artifacts.

Dong et al. developed an image‐based MAR method which utilizes the anatomical similarity in adjacent CT slices which are free from the metal artifacts (CT_ref_).^72^ Initially, a gamma map is calculated which is a weighted summation of the relative HU error and the distance error for each pixel between the CT_art_ slice and the relative artifact‐free CT slice. Then, the minimum value in the gamma map is used to identify and replace the artifact‐corrupted pixels on the CT_art_ slice by the corresponding pixels on the artifact‐free CT slice.

An MAR method which includes a sinogram precorrection (which is not described) and a hardware adaptation for proton therapy was proposed by Jin et al.^77^ The hardware adaptation includes an increase in the X‐ray energy from 120 kVp (standard energy) to 180kVp, and an increase in the triggering rate of data acquisition system (DAS)^88^ of the X‐ray detectors. In the end, the prelog iterative CT reconstruction method^89^ is applied to yield the resulting CT_cor_.

Miksys et al. studied image‐based MAR methods and a sinogram‐based MAR method to reduce the metal artifacts from and around implanted radioactive seeds which contain Ag and Ti for brachytherapy procedures.^74^ The image‐based MAR methods include simple threshold replacement (STR), 3D median filter, and the generation of a virtual sinogram. STR utilizes a HU thresholding technique to identify the seeds and bright spot artifacts,^90^ and correct them by replacing them with predefined HU value. The 3D median filter instead reduces the metal artifacts by smoothing the high voxel values which are identified through HU thresholding. In the virtual sinogram generation, first the metal on CT_art_ is segmented using thresholding and then CT_art_ and the segmented metal are forward projected. Subtraction of the metal projection from the CT_art_ projection resulted in a partial sinogram. Finally, through the application of interpolation and filtered back projection, the partial sinogram yields the CT_cor_. The sinogram‐based MAR method also utilizes similar steps, but instead of generating a virtual sinogram, it uses the sinogram from the CT scanner.

##### Evaluation of HU values retrieval and image quality

Research‐based MAR algorithms based on traditional image processing methods were evaluated for their ability to restore HU values and their ability to improve image quality of CT scans which will be used for RT applications. Table 5 summarizes the details of the corresponding publications.

**Table 5:** Summary of the studies which investigated HU restoration and image quality improvement of research‐based MAR algorithms for RT application which were based on traditional image processing methods.

Author	MAR	Images; No. of sample (n)	Metals	CT scans	ROI; [Reference HU value (Mean)]	Measurements	Results	Findings
Park et al.^79^	MRI‐based CT MAR	Clinical (H&N) & brain); (n = 2)	Simulated artifacts	(CT_art_ vs CT_ref_ ) & (CT_cor_ vs CT_ref_ )	Whole CT scan Brain; [Not reported] H&N; [ Not reported]	Absolute mean HU different	495 HU & 108 HU 370 HU & 92 HU	MRI‐based CT MAR improved the HU value measurement
Nielsen et al.^84^	kerMAR	Phantom Clinical (H&N); (n = 9)	Metal pins Dental implants	CT_art_ vs CT_cor_ (kerMAR) + CT_cor_ (O‐MAR) CT_cor_ (O‐MAR) vs CT_cor_ (kerMAR)	Soft tissue Bone Oral cavity Mandible Teeth	Differences in low/high frequencies in HU	Refer to figs. 6 & 7 in Ref. [84] Refer to fig. 6 & 7 in Ref. [84]	kerMAR can be used for restoration of the HU values
Liugang et al.^81^	MVCBCT & kVCT method	Phantom Clinical (H&N); (n = 1)	SS with different diameter Dental implant	CT_art_ & CT_cor_ & CT_ref_ (CT_art_ vs CT_ref_) & (CT_cor_ & CT_ref_)	Pixel value along horizontal & vertical lines on CT scan Whole CT Scan	HU Percentage HU difference	Refer to fig. 8 in Ref. [81] Refer to fig. 7 in Ref. [81]	Proposed MVCBCT & kVCT methods improved the accuracy of HU value measurements
Jeon et al.^82^	Hybrid sinogram‐based MAR	Phantom Clinical (H&N); (n = 3)	Ag, Sn, Cu & Fe Dental filling	CT_art_ (kVCT) vs CT_ref_, CT_art_ (MVCT) vs CT_ref_ & CT_cor_ vs CT_ref_ CT_art_ (MVCT) vs CT_ref_ & CT_cor_ vs CT_ref_ CT_cor_ vs CT_ref_	Non‐metal tissue; [Not reported] Metal inserts; [Not reported] Whole image; [Not reported]	Mean true density errors Visual quality	1.81%, 0.42% & 1.94% 0.48% & 0.30% Refer to fig. 9 in Ref. [82]	Hybrid sinogram‐based MAR can be used for image quality and HU values improvement
Kim et al.^76^	Additional tilted CT scan‐based MAR	Simulation Phantom Experimental phantom	Bilateral hip implant Dental implants	(CT_art_ vs CT_ref_) & (CT_cor_ vs CT_ref_)	Whole image; [Not reported]	Mean HU absolute percentage error (%)	62.98% & 8.52% 94.12 & 10.12%	Metal artifacts can be reduced by the proposed additional tilted CT scan‐based MAR
Dong et al.^72^	Image‐based MAR	Clinical (Hip); (n = 1) Clinical (H&N); (n = 1)	Simulated artifacts Dental fillings	CT_art_ & CT_cor_	Whole image; [Not reported]	Mean HU error	360 HU& 24HU 460 HU & 34 HU	Image‐based MAR improved the accuracy of measured HU values

H&N Head & Neck, Ti Titanium, SS Stainless Steel, Ag Silver, Sn Tin, Cu Copper, Fe Iron, kVCT kilovoltage CT, MVCT Megavoltage CT, CT_art_ CT scans with artifacts, CT_cor_ Corrected CT scans, CT_ref_ Reference CT scans, vs versus.

The proposed MRI‐based MAR algorithm by Park et al. was evaluated for its ability to improve the HU values.^79^ Simulated metal artifacts were introduced on clinical H&N and brain CT_ref_, and then the proposed MAR algorithm was applied to generate a CT_cor_. The comparison of CT_art_ and CT_cor_ with CT_ref_ showed a reduction in absolute mean HU difference from 495 HU to 108 HU in the brain, and from 370 HU to 92 HU in the H&N. The HU of CT_ref_ was not reported. Nielsen et al. evaluated their kerMAR method for image quality improvements.^84^ During the image quality evaluation, CT scans from a custom‐made veal shank phantom with metal inserts and clinical H&N CT scans with dental implants were used. In addition to kerMAR, also O‐MAR was applied to the CT scans for a comparison. On the scans of the phantom, kerMAR and O‐MAR reduced the metal artifacts similarly (*P > 0.05*); however, on the clinical CT scans kerMAR outperformed O‐MAR (*P < 0.01*). This difference can be explained by the fact that O‐MAR produces residual artifacts and by the fact that kerMAR uses corresponding MRI images as an external source of prior information. However, if the acquired CT and MRI scans are not aligned properly, the kerMAR method introduces new artifacts as well.

The MVCBCT‐ and kVCT‐based MAR method^81^ was evaluated for HU value improvement using an intensity‐modulated verification phantom (CIRS, Norfolk, VA, USA) with SS inserts and clinical H&N CT scans with dental implants. The study found that the proposed MAR method reprojected the HU values with high accuracy. The hybrid sinogram‐based MAR method by Jeon et al. was evaluated using a standard “cheese” phantom (Standard Imaging, Middleton, USA) with metal inserts made of Ag, tin (Sn), copper (Cu), and iron (Fe), and clinical H&N CT scans with dental implants.^82^ Application of the proposed method improved the accuracy of true densities and HU values for metal and non‐metal tissues (e.g., air, lung, and bone) on the phantom and the clinical scans, respectively. In addition to the HU value improvements, also CNR and SNR improved on the CT_cor_ after application of the hybrid sinogram‐based MAR in comparison with CT_art_.

The MAR method which requires the acquisition of an additional tilted CT scan^76^ was evaluated for its ability to improve HU values In an experimental study, a customized RANDO head phantom (The Phantom Laboratory Inc., NY, USA) with dental implants was used, while in a simulation study an XCAT numerical phantom^91^ with bilateral hip implants was used. The mean absolute percentage errors in HU values were calculated for CT_art_ and CT_cor_ in comparison with CT_ref_. This showed a reduction for CT_cor_ of up to 89% in the experimental study, and up to 86% in the simulation study. Dong et al. evaluated their image‐based MAR method on clinical H&N CT scans with dental fillings and on a CT scan of a pelvis with simulated hip implants.^72^ The mean HU error was calculated on both CT_art_ and CT_cor_ compared with CT_ref_ and it reduced from 360 HU to 24 HU in the H&N case, and from 460 HU to 34 HU in the pelvis case. Thus, the study concluded that the proposed image‐based MAR can be used to reduce the metal artifacts and improve HU for RT applications.

The proposed image‐based MAR methods and sinogram‐based MAR method for brachytherapy by Miksys et al. were applied on CT scans of a custom‐made agarose gel phantom and on clinical prostate CT scans with implanted seeds.^74^ The STR removed bright spot artifacts around the seeds, but failed to mitigate any dark streak artifacts. Although the 3D median filter successfully mitigated the white and dark streak artifacts, it blurred the resulting CT_cor_. The virtual sinogram and the raw sinogram methods reduced the streak artifacts, however, they introduced new artifacts on CT_cor_. The authors concluded that the image‐based MAR methods, especially the STR and 3D median filter, were more successful in reducing the artifacts in comparison with the sinogram‐based MAR.

##### Dosimetric impacts of traditional MAR algorithms

In addition to the improvement of HU and image quality, dosimetric impacts of research‐based traditional MAR algorithms were evaluated in several research studies. Table 6 describes the findings of these evaluations.

**Table 6:** Summary of the studies which investigated the dosimetric impact in RT of research‐based MAR algorithms based on traditional image processing.

Author	MAR	Images; No. of Samples (n)	Metal	Beam, Energy, and (Mode of therapy)	Dose calculation algorithm (TPS); [Dose measurement]	CT Scans	Calculation and Measurement	Results	Comments
Park et al.^79^	MRI‐based CT MAR	Clinical (H&N): & brain); (n = 2)	Simulated artifacts	Proton	N/A	(CT_art_ vs CT_ref_) & (CT_cor_ & CT_ref_)	Absolute mean ΔWET	1.7cm & 2mm 2.4cm & 2mm	The proposed method increased the proton range calculation accuracy
Nielsen et al.^84^	kerMAR	Phantom Clinical (H&N); (n = 9)	Metal pins Dental implants	Photon (6 MV) Electron (12 MeV) Proton (150 MeV)	AAA, (Eclipse™, Varian) Electron MC Proton CS	CT_cor_ (O‐MAR) vs CT_cor_ (kerMAR)	Depth/Range Absolute difference (Depth/Range) (Mean ± STD)	Refer to fig. 7 in Ref. [84] 0.7 ± 0.5 mm 1.3 ± 0.3 mm 1.8 ± 0.4 mm	kerMAR increased the particle range calculation accuracy
Liugang et al.^81^	MVCBCT & kVCT method	Phantom	SS	Photons (6 MV)	AAA, (Eclipse™, Varian)	(CT_art_ vs CT_ref_) & (CT_cor_ & CT_ref_)	Maximum dose errors (%) Through hole Through phantom & metal	5.1% & 11.8% 3.3% & 17.2%	The proposed method improved the dose calculation accuracy
Ziemann et al.^92^	ALIR	Phantom	SS	Photons, 6 MV (VMAT)	PB and AAA (Eclipse™, Varian) [ionization chamber]	(CT_art_ vs Measured) & (CT_cor_ vs Measured)	Maximum dose errors (%)	8.4% & 2.7%	ALIR reduced the errors in dose calculation
Dong et al.^72^	Image‐based MAR	Clinical (Hip); (n = 1) Clinical (H&N); (n = 1)	Simulated artifacts Dental fillings	Photons	N/A	(CT_art_ vs CT_ref_) & (CT_cor_ vs CT_ref_)	%GP failure	23.25% & 0.02%,	The proposed method increased the dose calculation accuracy
Odlozilikova et al.^93^	MDT	Clinical (Breast and lymph nodes); (n = 1), (Esophagus); (n = 2), (H& N); (n = 2), (breast);(n = 2), (Lung); (n = 2)	CIEDs	Photon (3D‐CRT, IMRT, VMAT, and SBRT)	Eclipse™, Varian	CT_art_ vs CT_cor_	The greatest dose error to ECIDs	>3% of total calculated dose	The application of MDT reduced the dose to the cardiac devices as OARs.
Aziz et al.^73^	Sinogram‐based MAR	Phantom	Dental amalgam	Single‐electron beam (BEAMnrc), 9MeV	MC, DOSXYZnrc source code	CT_art_ vs CT_cor_	Dose error uncertainties at d80	56% & 2%	This MAR in combination with MC dose calculation reduced the dose calculation uncertainties
Jin et al.^77^	MAR with hardware adaptation	Clinical (Hip); (n = 1)	Simulated bilateral Ti implants	Proton	N/A	(CT_art_ vs CT_ref_) & (CT_cor_ & CT_ref_)	Mean ΔWET	7mm & <1mm	The proposed MAR reduced the proton range errors

Ti Titanium, SS Stainless Steel, CRT Conformal Radiation Therapy, IMRT Intensity‐Modulated Radiation Therapy, VMAT Volumetric‐Modulated Arch Therapy, AAA Analytical anisotropic algorithm, PB Pencil Beam, MC Monte‐Carlo, CT_art_ CT scans with artifacts, CT_cor_ Corrected CT scans, CT_ref_ Reference CT scans, vs versus, ECIDs Electronic cardiac implantable devices, ΔWET Error in water equivalent thickness, %GP Gamma passing rate, N/A Not available.

The MRI‐based MAR algorithm^79^ was evaluated to assess its ability to improve the proton range error (ΔWET) for proton therapy applications. On clinical CT scans of a brain and H&N, the application of this proposed method improved the absolute ΔWET from 2.4 cm in brain and 1.7 cm in H&N to less than 2 mm for both cases. In a study by Nielsen et al.,^84^ the performance of kerMAR was studied and compared with O‐MAR for dosimetric impact in photon, electron, and proton therapy. In the phantom evaluation, a depth/range calculation on CT_cor_ for the proton, photons, and electrons did not show any significant difference (*P > 0.05*) between the O‐MAR and kerMAR. However, an evaluation of the absolute particle range difference on clinical CT_cor_ showed that kerMAR significantly (*P < 0.001*) improved for electron beams nearly by 1 mm and proton beams by 2 mm in comparison with O‐MAR. However, the photon beam evaluation showed an insignificant difference in depth/range calculations on CT_cor_.

In a MVCBCT‐ and kVCT‐based MAR study,^81^ a custom‐made phantom with a SS metal rod containing an air‐filled hole in its center was CT scanned. The MAR method was applied to generate a CT_cor_ from CT_art_. Subsequently, dose calculations were performed on CT_art_, CT_cor_, and CT_ref_ using a single irradiation field in two instances, one crossing the hole and the other one crossing only the phantom and the metal. The calculated maximum percentage of dose error through the hole was 5.1% on CT_cor_ and 11.8% on CT_art_ in comparison with CT_ref_. The dose error through the phantom and the metal was 3.3% on CT_cor_ and 17.2% on CT_art_. So, this seems to imply that the calculated dose on the CT_cor_ after application of this MAR method was improved and better approximated the dose distribution on CT_ref_.

ALIR was studied by Ziemann et al.^92^ To simulate a pelvis with bilateral hip prostheses, a polymethylmethacrylate phantom with two SS rods was CT scanned. Then, ALIR, linear interpolation (LI), and density correction MAR methods were applied to create corresponding CT_cor_. Next, a PTV was chosen at the center of the phantom between the metal implants and dose calculations were performed on CT_cor_. Dose errors were computed between the calculated doses and respective measured doses using ion chambers. These dose errors were 2.7% for ALIR, 3.2% for LI, and 4.1% for the density correction. The CT_art_ showed a higher dose error of 8.4%. However, calculated dose to the simulated rectum as an OAR was higher for the ALIR method than for the LI method. The study concluded that ALIR can be applied for successful RT planning.

The image‐based MAR^72^ was assessed for the dosimetric impact on clinical H&N CT scans with dental fillings and on a CT scan of a pelvis with simulated hip implants. The calculation of gamma failure rates for a 3%/3 mm gamma criterion on CT_art_ and CT_cor_ compared to CT_ref_ resulted in 23.25% and 0.02%, respectively. In clinical studies, Odlozilikova et al. evaluated the MDT algorithm for dose error to cardiac implantable electronic devices (CIEDs) as an OAR while planning to irradiate the adjacent target tissues.^93^ The total planned dose to the PTV was used to calculate the dose error to the CIEDs on CT_art_ and CT_cor_. The largest dose error for the CIEDs was on the CT_art_, and it was more than 3% of the total planned dose. Hence, the application of MDT significantly improved RT planning and reduced the dose error to the CIEDs.

Aziz et al. evaluated dose calculation errors induced by a dental amalgam in a custom‐made phantom experiment.^73^ An in‐house developed LI was applied to correct the metal artifacts, and a simulated electron beam was used to evaluate the dosimetric errors between the dose calculation plans on CT_art_ and CT_cor_. Uncertainties in the calculated dose error reduced from 46% on CT_art_ to 2% on CT_cor_ at d_80_.^94^ For this reason, it was suggested to apply the proposed method to improve the dose calculation accuracy in electron beam therapy. In another study, clinical CT scans of a hip with simulated Ti implants were used for dose calculation on CT_art_, CT_cor_ after application of MAR method by Jin et al.,^77^ and CT_ref_. The proposed MAR method reduced the proton mean ΔWET from 7 mm on CT_art_ to less than 1 mm on CT_cor_ in comparison with CT_ref_.

#### 3.B.2 Deep learning‐based MAR algorithms

Few research studies using deep learning‐based methods to reduce metal artifacts on CT scans for RT applications were found in literature. These deep learning MAR approaches are either based on 2D residual learning‐based convolutional neural networks (CNNs)^95^, ^96^ or 2D cycle generative adversarial networks (GANs).^97^

**Fig. 5:**
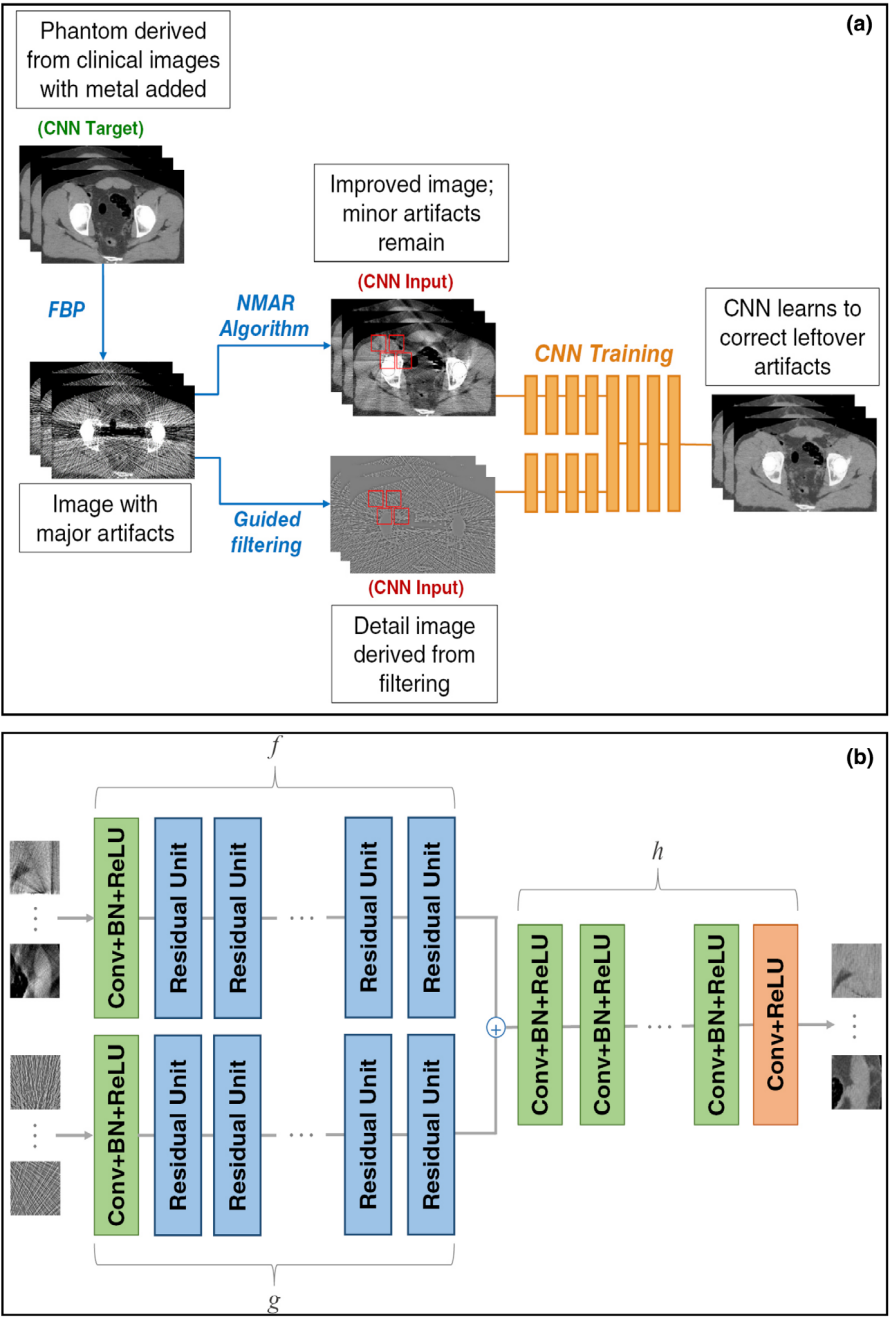
Overview of the deep learning approach proposed by Gjesteby et al.^96^ (a) The proposed dual‐stream deep network for metal artifacts reduction. (b) Details of the CNN training of (a). (Reproduced from Gjesteby et al.^96^. © Institute of Physics and Engineering in Medicine. Reproduced by permission of IOP Publishing. All rights reserved).

A dual‐stream deep network was proposed by Gjesteby et al. which utilizes the residual learning‐based CNNs (see Fig. 5).^96^ This network requires paired data, CT_art_ and CT_ref_, during the artifact reduction. First, a custom implementation of normalized MAR (NMAR)^98^ and guided filtering^99^ are applied to the artifact corrupted CT slice to reduce the metal artifacts and preserve the edge information of a CT slice, respectively. Subsequently, patches [indicated by ‘red’ squares in Fig. 5(a)] are extracted from these CT scans and are input into two identical parallel streams *f* and *g* [Fig. 5 (b)]. Each of these parallel streams starts with an initial parameter layer and is followed by 20 residual units. This network structure is referred to as DestreakNet.^100^ Each residual unit consists of two convolutional layers (Conv) followed by batch normalization (BN) and a rectified linear unit (ReLU) [Fig. 5(b)]. The residual units help the network to learn the residual errors and generate respective feature maps. Finally, the feature maps from these streams are summed and passed together through *h* [Fig. 5(b)] to produce patches with metal artifact correction. The stream *h* contains another eight parameter layers and a final layer without the BN. During training, DestreakNet is optimized using a mean square error (MSE) loss function and perceptual loss function.^101^ The MSE loss function is used to calculate the pixel‐by‐pixel error between the output and the target, while the perceptual loss function mitigates oversmoothing which can result from the MSE loss function.

A similar study, also using a residual learning‐based artifact reduction CNN (RL‐ARCNN) and paired data for artifact reduction, was proposed for brachytherapy applications by Huang et al.^95^ This network architecture consists of a CNN, and ReLU is applied to increase the nonlinearity of the input layer. In subsequent convolutional layers, BN is applied between the convolution and ReLU to achieve faster training. This network was trained for MAR through the residual learning method as proposed by He et al.^102^

The main limitation of the above both networks, dual‐stream deep network and RL‐ARCNN, is that they both need paired data for training. To overcome this challenge, Koike et al. developed a deep learning‐based MAR (DL‐MAR)^97^ using CycleGANs in which U‐Net and PatchGAN architecture of the pix2pix model^103^ are used as generator and discriminator, respectively. The generator synthesizes CT_cor_ from CT_art_ and vice versa, while the discriminator aims to distinguish the synthetic CT_cor_ scans as fake. The authors made the following modifications from the paper published by Isola et al.^103^: an instance normalization layer^104^ replaced the BN layer in the generator, the kernel size of convolutional layer was set to 4X4 with a stride of 2 instead of the maximum pooling filter, the image size was expanded by using the upsampling filters and one filter convolution layer was applied followed by a tanh activation function as the last filter. For all the other layers, a leaky rectified linear unit (LeakyReLU) with a slope of 0.2 was used as an activation function. In this proposed method, the CT_art_ is translated into CT_cor_ using adversarial loss, cycle consistency loss,^105^ and identity map loss which were introduced to regularize the generator during the conversion.

##### Evaluation of HU values restoration and image quality

The proposed dual‐stream deep network, RL‐ARCNN, and DL‐MAR were evaluated for their ability to restore HU values and image quality. The findings of these studies are summarized in Table 7.

**Table 7:** Summary of the studies which investigated the restoration of HU values and image quality of deep learning‐based MAR algorithms for RT application.

Author	MAR	Images; No. of CT slices /samples (n) [for training & for MAR]	Metals	CT scans	ROI	Measurements	Results	Comments
Gjesteby et al.^96^	Dual‐stream CNN with residual learning	Phantom derived clinical (Hip & Spine); [42 & 08]	Simulated Ti	(CT_art_ vs CT_ref_ ) & (CT_cor_ vs CT_ref_ ) CT_ref,_ CT_art_ & CT_cor_	Whole image Hip Spine Near to metal implants on Hip Spine	SSIM PSNR in dB Mean HU	0.2382 & 0.8262 0.6930 & 0.8723 9.1830 & 22.1685 15.5450 & 21.8480 80.56, 2.25 & 94.26 94.27, 121.73 & 89.18	The proposed method is highly effective to reduce metal artifacts
Huang et al.^95^	RL‐ARCNN	Clinical (Prostate); [550 &550]	Simulated & real artifacts	(CT_cor_ vs CT_ref_ ) & (CT_art_ vs CT_ref_ )	Whole image	PSNR in dB	38.09 & 25.38	RL‐CNN eliminates the metal artifacts better than conventional image processing‐based MAR methods
Koike et al.^97^	Cycle GAN	Clinical (H&N); [(n = 92) & (n = 15)]	Dental fillings	(CT_art_ vs CT_ref_ ) & (CT_cor_ vs CT_ref_)	Oral cavity	Artifact index [mean ± STD]	13.2 ± 4.3 & 267.3 ± 113.7	The proposed method reduced the metal artifacts from H&N CT scans

H&N Head & Neck, Ti Titanium, CT_art_ CT scans with artifacts, CT_cor_ Corrected CT scans, CT_ref_ Reference CT scans, vs versus, SSIM Structural similarity, PSNR Peak Signal‐to‐Noise Ratio.

The dual‐stream deep network with residual learning^96^ was evaluated on synthesized data. Two kinds of voxelized phantoms were created from volumetric data of the pelvis and spinal regions from the visible human project.^106^ CT scans of these voxelized phantoms were simulated by an industrial CT simulation software^107^ with and without a Ti implants to mimic hip prostheses and spinal implants. Then, the proposed MAR algorithm was trained to generate CT_cor_ from these CT scans. The authors measured the mean HU near the metal implant on CT_ref_, CT_art,_ and CT_cor_ and the results were as follows: 80.56, 2.25, and 94.26 HU in the hip case and 94.27, 121.73, and 89.18 HU in the spine case, respectively. Also, calculations of SSIM and PSNR were performed for CT_art_ and CT_cor_ in comparison with CT_ref_. For the SSIM calculation, a value of 1 indicates the highest similarity between the compared CT scans, while 0 indicates the lowest similarity. The calculated SSIM were 0.2382 for CT_art_ and 0.8262 for CT_cor_ for the hip case, and 0.6930 for CT_art_ and 0.8723 for CT_cor_ for the pelvis case. In addition, the calculated PSNR were 9.1830 dB for CT_art_ and 22.1685 dB for CT_cor_ for the hip case, and 15.5450 dB for CT_art_ and 21.8480 dB for CT_cor_ for the pelvis case. These results indicate that the proposed dual‐stream deep network with residual learning can be used to reduce the metal artifacts for RT applications. However, this proposed MAR algorithm heavily depends on the performance of NMAR, used for data preprocessing.

For the brachytherapy application, RL‐ARCNN^95^ was evaluated. During the training, paired clinical cervical CT scans which include CT_ref_ and simulated artifacts on CT_ref_, and CT scans with residual artifacts were used. The CT scans with residual artifacts were obtained from the difference between CT_ref_ and CT_ref_ with simulated artifacts. During the MAR evaluation, clinical cervical CT scans with implanted seeds were used (CT_art_). The calculated PSNR was 38.09 dB for CT_cor_ after application of RL‐ARCNN and 25.38 dB for CT_art_. So, the proposed method seems to reduce the metal artifacts.

DL‐MAR was studied on CT scans with dental implants by Koike et al.^97^ The Adam optimizer was used to train the network using unpaired clinical H&N CT scans with (CT_art_) and without (CT_ref_) the dental implant. The calculation of the artifact index^22^ (mean ± STD) between CT_cor_ and CT_art_ resulted in significant differences (*P* < 0.001), and it was 13.2 ± 4.3 on CT_cor_ and 267.3 ± 113.7 on CT_art_. Therefore, this proposed MAR algorithm can be used to reduce the metal artifacts for RT applications. However, the authors stated that the proposed MAR algorithm was trained and modified for the H&N CT scans and can only be applied on these scans.

##### Dosimetric impact of deep learning‐based MAR algorithms

During the evaluation of DL‐MAR^97^ for RT, clinical H&N CT scans with dental implants were used. A density correction method using water density (1.0 g cm^‐3^) was used for the comparison. IMRT planning with 6MV photons was performed using seven fields. To calculate the dose for the oral cavity on CT_art_ and CT_cor_, the AAA algorithm was used. DAH was used to evaluate the calculated doses on CT_cor_ after application of DL‐MAR and CT_art_ in comparison with the CT_cor_ after application of density correction. The maximum dose differences were −2.4% on CT_cor_ after application of DL‐MAR and −7.2% on CT_art_. Moreover, very small dosimetric differences were found between the calculated doses on CT_cor_ after application of DL‐MAR and CT_cor_ after application of density correction. However, the plan dose distribution on CT_cor_ after application of density correction may not reflect the actual dose distribution.

## 4 DISCUSSION

Table 8 lists the strength and weakness of the proposed or evaluated MAR methods for RT applications. Commercial MAR algorithms are used in clinical environments to improve the treatment delivery in wide range of cases. These algorithms are self‐optimized (automatic) and do not require skills from an operator. On the other hand, in density correction methods, metal artifacts are identified by an expert and corrected by appropriate density overrides. Among the investigated research‐based traditional MAR algorithms, the application of kerMAR reduced the metal artifacts more efficiently for H&N cases than O‐MAR did. Even though ALIR improved the dose calculation accuracy more than the density correction method, it failed to reduce the planned radiation dose to certain OARs. MRI‐based CT MAR^79^ requires manual selection of CT scans for artifact correction. Most of the proposed MAR algorithms based on traditional image processing such as MVCT‐based MAR, additional tilted CT scan‐based MAR, and ALIR were performed better than LI or/and NMAR for artifact correction. Among the deep learning‐based MAR algorithms, DL‐MAR does not require paired data for artifact correction, and it provided similar results for dose calculation when compared with the water density override.

**Table 8:** Strengths and weaknesses of proposed and/or investigated MAR methods for RT applications.

MAR methods		Strength	Weakness
Commercial MAR methods	O‐MAR, iMAR, Smart MAR & SEMAR ^35^, ^41^, ^42^, ^43^, ^44^, ^45^, ^46^, ^47^, ^48^, ^49^, ^50^, ^51^, ^52^, ^53^, ^54^, ^55^, ^56^, ^57^, ^59^, ^62^, ^63^	Standard methods and are used routinely in the clinic. Applicable on a wide range of clinical cases for RT applications.	Incomplete removal of artifacts with HU errors. Induce new artifacts. Scanner specific.
	Density correction methods ^38^, ^67^, ^68^, ^71^	Available in TPS.	Manual methods. Operator needs specialized knowledge and experience for artifact identification and density overrides.
Research‐based traditional MAR algorithms	Image based MAR^72^	Do not use sinogram data	Semi‐automatic
	MDT^93^	Reduces the dose to CIEDs during various RT procedures.	May requires long processing time
	Sinogram‐based MAR^73^	Reduces the dose errors from amalgam in H&N RT.	Applicable for minor streaks artifacts
	STR, 3D median filter and Sinogram‐based correction^74^	STR removes the spot artifacts. 3D median filter and sinogram‐based corrections reduce the dark and white streaks.	STR and 3D median filter use predefined HU values and are applicable only for brachytherapy. Sinogram‐based corrections induce new artifacts.
	Additional tilted CT scan‐based MAR^76^	Reduces the HU errors better than LI and NMAR.	Additional radiation burden.
	ALIR^92^	Improves the dose calculation accuracy more than density correction method and LI do.	Increases the calculated doses for OAR.
	MAR with hardware adaptation^77^	Addresses the photon starvation during the artifact reduction.	Modified CT image acquisition in comparison with the standard.
	MRI‐based CT MAR^79^	Does not require sinogram and threshold‐based tissue classification.	Requires aligned MRI & CT scan. Semi‐automatic.
	kerMAR^84^	Reduces the metal artifacts better than O‐MAR in H&N cases.	Requires aligned MRI & CT scan. Applicable only for H&N case.
	MVCBCT & kVCT method^81^	Performs better than LI and NMAR for artifact reduction.	Requires MVCBCT scan.
	Hybrid sinogram‐based MAR^82^	Performs better than LI for artifact reduction.	Requires MVCT scan.
Deep learning‐based MAR algorithms	Dual‐stream CNN with residual learning^96^	Reduces the remaining metal artifacts on CT scans after NMAR application.	Requires paired data and depends on the performance of NMAR
	RL‐ARCNN^95^	Does not require sinogram data.	Needs paired data.
	DL‐MAR^97^	Does not require paired data. Comparable performance to density correction for accuracy in dose calculation.	Applicable only in H&N cases.

The application of MAR methods on CT scans for RT applications was summarized in this review. Commercial MAR methods are utilized in clinical environments to correct the metal artifacts and improve the dosimetric accuracy in RT. However, residual artifacts and/or creation of new artifacts which may negatively impact in RT planning are often identified. Also, planned dose distributions on CT scans after application of TPS‐based density correction methods show large‐dose discrepancies compared to delivered dose. Thus, the performance of commercial MAR methods is not always completely satisfactory in RT application.

Among the research‐based MAR methods, some of the MAR methods which are based on traditional image processing require an additional CT or aligned MRI scan which may result in extra radiation burden for the patient, or which may not be accessible. Only few studies compared the efficiency of the proposed MAR methods with the commercial MAR methods for RT applications. In addition, the ability of the proposed deep learning‐based MAR algorithms to correct artifacts depends on the amount of CT scans from a specific anatomy which are used to train them. Thus, they are typically optimized to reduce the metal artifacts for a specific anatomy. Furthermore, their dosimetric impacts for RT applications were not extensively evaluated.

The commercial MAR methods and research‐based MAR methods still have a limitation in metal artifact correction and/or dose improvements for RT applications. Their ability of the metal artifact corrections mainly depends on the anatomical region of CT scans which are corrupted by the metal artifacts from a specific implanted metal. The patterns and severity of the metal artifacts from each of implants are unique and different. The traditional MAR methods have difficulties in recognizing the patterns of the metal artifacts, but deep learning approaches can efficiently handle this complicated challenge. Also, newly induced artifacts were identified on CT scans after application of MAR algorithms which utilize the sinogram for the metal artifacts reduction. The deep learning‐based MAR algorithms often work on reconstructed CT scans and, thus, the artifacts‐corrected CT scans are free from these induced artifacts. Moreover, recent developments of MAR algorithms which utilize deep learning, for example, Cycle GANs do not require paired clinical CT scans for artifact reduction. Therefore, developing a MAR method while targeting a specific pattern of metal artifacts and a specific anatomical structure using a deep learning approach will be a promising solution. This method should be explored further and then evaluated for the RT applications.

## CONFLICTS OF INTEREST

The authors declare that they have no conflict of interest.

## Authors Contributions

Sathyathas Puvanasunthararajah was involved in conceptualization, data extraction, and drafting of the manuscript. Davide Fontanarosa and Marie‐Luise Wille were involved in conceptualization, reviewing, and supervision. Saskia M. Camps was involved in conceptualization, and checked the accuracy of data extraction, reviewing, and supervision.
